# Sirtuins in Central Nervous System Tumors—Molecular Mechanisms and Therapeutic Targeting

**DOI:** 10.3390/cells14141113

**Published:** 2025-07-19

**Authors:** Agnieszka Nowacka, Martyna Śniegocka, Maciej Śniegocki, Ewa Aleksandra Ziółkowska

**Affiliations:** 1 Department of Neurosurgery, Collegium Medicum in Bydgoszcz, Nicolas Copernicus University in Toruń, ul. Curie Skłodowskiej 9, 85-094 Bydgoszcz, Poland; 2 Department of Anatomical, Histological, Forensic & Orthopedic Sciences, Section of Histology & Medical Embryology, Sapienza University of Rome, Via A. Scarpa, 14–16, 00161 Rome, Italy; 3 Department of Pediatrics, School of Medicine, Washington University in St. Louis, St. Louis, MO 63110, USA

**Keywords:** sirtuins, SIRT, CNS tumors, central nervous system tumors, glioblastoma, GBM, glioma, GSCs, glioblastoma stem cells, ATRT, medulloblastoma, oncogene, tumor suppressor, cell metabolism, NAD+-dependent deacetylases, histones

## Abstract

Sirtuins (SIRTs), a family of NAD+-dependent enzymes, play crucial roles in epigenetic regulation, metabolism, DNA repair, and stress response, making them relevant to glioma biology. This review systematically summarizes the molecular mechanisms and context-specific functions of SIRT1–SIRT7 in central nervous system tumors, with particular focus on gliomas. SIRT1, SIRT3, SIRT5, and SIRT7 are often overexpressed and promote glioma cell proliferation, stemness, therapy resistance, and metabolic adaptation. Conversely, SIRT2, SIRT4, and SIRT6 generally exhibit tumor-suppressive functions by inducing apoptosis, inhibiting invasion, and counteracting oncogenic signaling. Preclinical studies have identified several sirtuin modulators—both inhibitors and activators—that alter tumor growth, sensitize cells to temozolomide, and regulate pathways such as JAK2/STAT3, NF-κB, and mitochondrial metabolism. Emerging evidence positions sirtuins as promising targets for glioma therapy. Future studies should evaluate sirtuin modulators in clinical trials and explore their potential for patient stratification and combined treatment strategies.

## 1. Introduction

**Figure 1:**
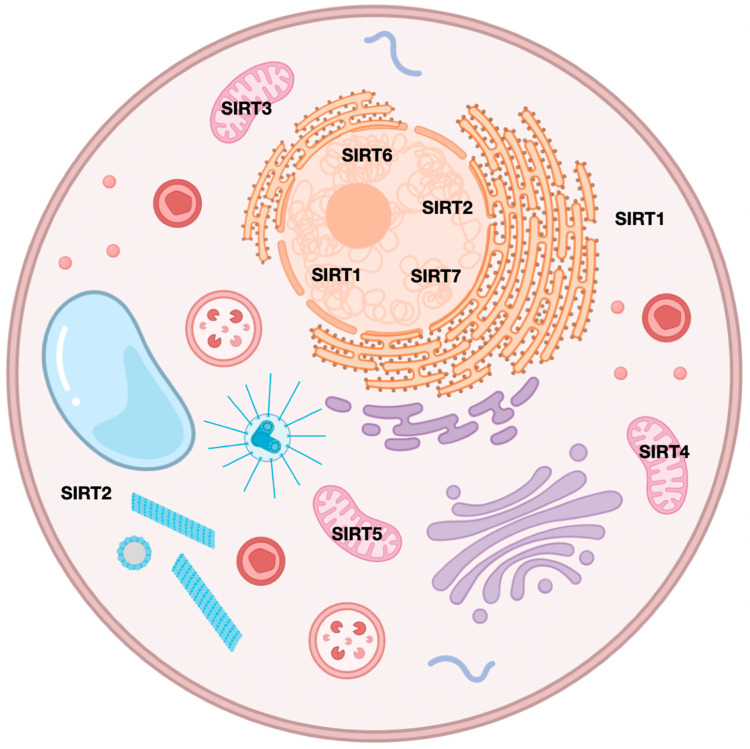
Localization of sirtuins in cellular components.

Sirtuins (SIRTs—silent information regulators), a family of seven proteins (SIRT1–SIRT7), are class III histone deacetylases involved in various cellular processes, including energy metabolism, DNA (deoxyribonucleic acid) repair, and aging [1]. In cancer, they exhibit dual roles, acting as tumor suppressors or oncogenes, depending on the cancer type and cellular context [1,2,3]. Sirtuins are localized in different cellular compartments: SIRT1 and SIRT2 are found in the nucleus and cytoplasm; SIRT3, SIRT4, and SIRT5 in the mitochondria; and SIRT6 and SIRT7 primarily in the nucleus (Figure 1) [4,5].

**Figure 2:**
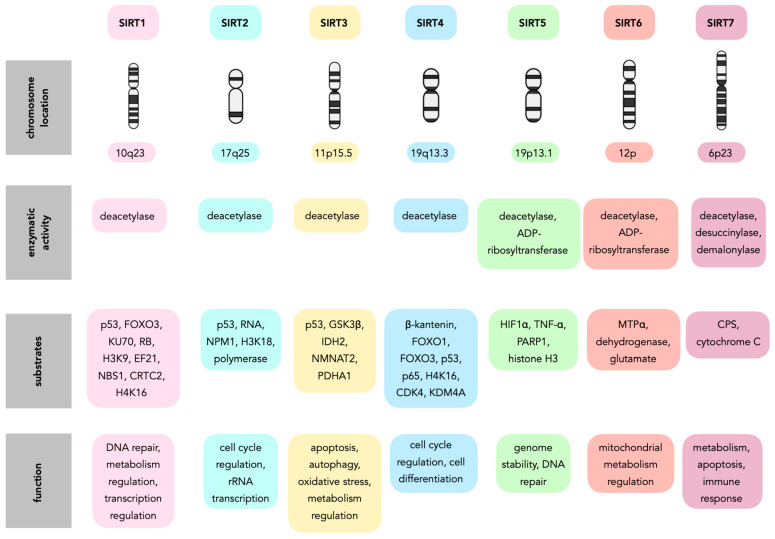
Sirtuins characteristics.

This localization influences their functions, with mitochondrial sirtuins mainly involved in energy metabolism and nuclear sirtuins in transcriptional regulation and DNA repair (Figure 2) [6,7]. For instance, SIRT1 has been shown to promote cancer progression by deacetylating and activating oncogenic transcription factors, while SIRT5 has been implicated in tumor suppression by regulating mitochondrial metabolism [4,8].

## 2. SIRT1

SIRT1 (sirtuin 1), localized in the nucleus and cytoplasm, is a member of the sirtuin protein family that exhibits a conserved molecular architecture featuring a unique N-terminal domain and C-terminal domain that surround a catalytic core [9,10,11]. These flanking domains mediate SIRT1-specific functions, such as homo-oligomerization and activation by small molecules like resveratrol [10,11,12]. Notably, the N-terminal domain is largely disordered, contributing to the protein’s extended conformation [10,11]. Despite their disordered nature, both termini enhance SIRT1’s deacetylase activity, indicating a regulatory role [10,11]. The catalytic core, highly conserved among sirtuin family members, is essential for SIRT1’s enzymatic activity, which involves removing acetyl groups from lysine residues on target proteins [10,13]. SIRT1-mediated deacetylation impacts multiple biological processes, including cellular senescence, apoptosis, sugar and lipid metabolism, oxidative stress, and inflammation [9,13,14].

SIRT1 plays a significant role in epigenetic regulation by deacetylating histones, which leads to chromatin remodeling and the regulation of gene expression, closely associated with aging and age-related pathologies [15,16,17]. In metabolic regulation, SIRT1 deacetylates transcription factors and coactivators like PGC-1α (peroxisome proliferator activated receptor gamma coactivator-1 alpha), influencing mitochondrial biogenesis and oxidative metabolism as well as insulin sensitivity and glucose metabolism, making it a target for treating metabolic diseases [16,18,19,20,21]. Additionally, SIRT1 regulates stress responses by deacetylating proteins involved in DNA repair, such as Ku70, and modulating pro-apoptotic factors like BAX (Bcl-2-associated X protein) [22]. It also promotes cell survival by inhibiting apoptosis and necroptosis [23,24]. In cancer, SIRT1 has a dual role, exhibiting both tumor-promoting and tumor-suppressive activities, regulating the acetylation status of tumor suppressors like p53 and oncogenic factors like NF-κB (nuclear factor kappa B) [16,19]. Moreover, SIRT1 is involved in reproductive and endocrine functions, including ovarian function, spermatogenesis, and hormonal pathways like the hypothalamic–pituitary–gonadal axis [24,25,26,27]. In the central nervous system, SIRT1 offers neuroprotection against neurodegenerative diseases like Parkinson’s by reducing oxidative stress, inflammation, and mitochondrial dysfunction [28,29,30,31,32].

SIRT1’s versatile biological functions are primarily mediated through its deacetylase activity, which targets both histone and non-histone proteins. Its mechanisms of action include the following:

- Histone deacetylation—SIRT1 deacetylates histones, leading to chromatin condensation and the regulation of gene expression, a process critical for aging and metabolic regulation [15,16,17].
- Non-histone protein deacetylation—In addition to histones, SIRT1 deacetylates a wide array of non-histone proteins, including transcription factors like p53 (tumor protein p53), FOXO (forkhead box O), and NF-κB, as well as DNA repair proteins like Ku70 and metabolic regulators like PGC-1α [16,18,24,33,34].
- Modulation of signaling pathways—SIRT1 also regulates key signaling pathways such as the insulin/IGF-1, AMP-activated protein kinase (AMPK), and NF-κB pathways, which are involved in metabolism, stress responses, and inflammation [14,19,22,33].
- Interactions with transcription factors—Furthermore, SIRT1 interacts with transcription factors to regulate gene expression; for example, it deacetylates p53, modulating its activity in cell cycle arrest and apoptosis [16,24,34].
- Mitochondrial regulation—Finally, SIRT1 promotes mitochondrial function by deacetylating PGC-1α, enhancing mitochondrial biogenesis and oxidative phosphorylation [21,35,36].

SIRT1 activity and expression are intricately regulated by several factors. NAD+ availability is crucial, as SIRT1’s enzymatic activity depends on NAD+ levels, which fluctuate with metabolic states; caloric restriction increases NAD+ levels, activating SIRT1 [19,22,37]. Small-molecule activators like resveratrol, found in red wine, and other natural compounds such as fisetin, quercetin, and curcumin, can modulate SIRT1 activity [20,38,39]. Conversely, inhibitors like nicotinamide can suppress SIRT1 activity [38]. Post-translational modifications, including phosphorylation, ubiquitination, and sumoylation, also regulate SIRT1’s activity, localization, and stability [10,33]. Caloric restriction induces sirtuin 1 expression, promoting longevity and metabolic health, mediated by AMPK activation and suppression of insulin/IGF-1 (insulin-like growth factor 1) signaling [19,22]. Furthermore, SIRT1 expression and activity tend to decline with age, contributing to age-related diseases such as cancer, neurodegeneration, and cardiovascular disorders [14,17,30,31,35].

SIRT1 shuttles between the cytoplasm and the nucleus, where it deacetylates histones and non-histone proteins involved in a plethora of cellular processes, including survival, growth, metabolism, senescence, and stress resistance [40]. It exhibits broad expression across various tissues, including the liver, muscle, adipose tissue, brain, and endothelium, influencing diverse physiological processes [14,21,37]. Notably, its expression is particularly high in the hypothalamus, where it plays a crucial role in regulating energy metabolism and circadian rhythms [2,21,28]. In immune cells, SIRT1 modulates inflammatory responses by deacetylating NF-κB and other pro-inflammatory factors [14,33,41].

### SIRT1 in CNS Tumors

SIRT1 is aberrantly localized in the cytoplasm of glioma cells, unlike its typical nuclear localization in normal cells [42]. This mislocalization is associated with altered cell proliferation and oxidative stress responses in glioma cells [42,43]. In glioma cells, SIRT1’s impact on cell proliferation differs from that in normal astrocytes. Brow et al. indicate that inhibiting SIRT1 with nicotinamide significantly reduces cell proliferation in glioma cells to a greater extent than in normal astrocytes [42]. Conversely, activating SIRT1 with resveratrol increases cell proliferation, suggesting a differential response between glioma and normal cells [42]. Regarding the oxidative stress response, nicotinamide rescues normal astrocytes from oxidative stress induced by hydrogen peroxide (H_2_O_2_), but it does not have the same protective effect on glioma cells, indicating a context-dependent role of SIRT1 in managing oxidative stress [42].

Li et al. indicated that SIRT1 is highly expressed in glioma tissues compared with adjacent non-tumor tissues, suggesting its involvement in glioma development [44]. Silencing SIRT1, achieved using siRNA, significantly inhibits the viability and invasion of the glioma cell lines U87 and U251, indicating its role in promoting glioma cell growth and spread [44]. The study also found that silencing SIRT1 increases epithelial markers and decreases mesenchymal markers (fibronectin and vimentin), suggesting that SIRT1 promotes EMT (epithelial–mesenchymal transition) in glioma cells, facilitating their invasive capabilities [44]. These findings suggest that SIRT1 supports glioma cell viability and invasion and influences the EMT process, making it a potential therapeutic target [44].

Chen et al. found that SIRT1 is overexpressed in glioma tissues and cell lines compared with normal brain tissues [43]. Higher levels of SIRT1 expression are associated with poorer overall survival in glioma patients [43]. Inhibiting SIRT1 significantly reduces the proliferation of glioma cells and makes these cells more sensitive to temozolomide (TMZ) [43]. SIRT1 inhibition leads to an increase in reactive oxygen species levels in glioma cells, which enhances the sensitivity of glioma cells to TMZ treatment [43]. Tumors derived from SIRT1-inhibited glioma cells show reduced growth compared with control tumors [45]. These findings indicate that SIRT1 is essential for glioma tumor growth and chemoresistance, making it a potential therapeutic target for glioma treatment.

Quantitative imaging of SIRT1 expression and activity in a rat model of intracerebral glioma has been successfully demonstrated using 2-[18F]BzAHA ([18F]-2-fluorobenzoylaminohexanoicanilide), a radiotracer for PET/CT/MRI (Positron Emission Tomography/Computed Tomography/Magnetic Resonance Imaging) [46]. The standard uptake values of 2-[18F]BzAHA in 9L tumors showed significant accumulation of the radiotracer in the tumor compared with normal brain tissue [46]. Histological analyses validated these imaging results, revealing heterogeneous upregulation of SIRT1 in the tumor parenchyma, particularly in hypoxic and peri-necrotic regions [46]. Pharmacologic inhibition of SIRT1 with EX-527 resulted in a significant reduction in the SUV and distribution volume of 2-[18F]BzAHA in the tumors, indicating effective inhibition of SIRT1 activity [46]. This suggests that PET/CT/MRI imaging with 2-[18F]BzAHA can be translated into clinical settings for selecting glioma patients who may benefit from SIRT1-targeted therapies and for monitoring the pharmacodynamics of SIRT1 inhibitors [46].

In their study, Tian et al. reported that SRT2183, a SIRT1 activator, effectively reduces the growth of glioma cells in a dose-dependent manner [47]. This reduction in cell viability has been observed in various glioma cell lines, including LN229, SF539, SF767, and U87MG [47]. SRT2183 induces cell cycle arrest and apoptosis in glioma cells, increasing the number of cells in the G1 phase of the cell cycle while decreasing those in the S and G2 phases [47]. A significant finding is that SRT2183 triggers endoplasmic reticulum stress in glioma cells, with elevated markers of ER stress in cells treated with SRT2183 [47]. The growth-inhibitory effects of SRT2183 were significantly reduced when cells were pre-treated with an ER (endoplasmic reticulum) stress inhibitor, highlighting the role of ER stress in its mechanism of action [47]. While SRT2183 also induces autophagy, targeting autophagy did not significantly affect its growth-inhibitory effects, suggesting that autophagy may not be the primary mechanism through which SRT2183 exerts its effects [47]. Overall, SIRT1’s modulation may influence glioma cell survival and proliferation, and SRT2183 has potential as a therapeutic agent against glioma by inducing growth inhibition and apoptosis, primarily through the activation of ER stress pathways [47].

A study by Yao et al. has identified sirtuin-1 as a significant prognostic factor in glioblastoma, with high levels of SIRT1 mRNA expression associated with better clinical outcomes [48]. A study identified Comp 5 (small-molecule compound F0911–7667) as an effective and selective activator of SIRT1, which induces autophagic cell death in glioblastoma cells via the AMPK-mTOR-ULK (AMP-activated protein kinase–mammalian target of rapamycin–Unc-51-like kinase) complex pathway [48]. Comp 5 also triggers mitophagy via the SIRT1-PINK1-Parkin (sirtuin 1–PTEN-induced kinase 1–Parkin RBR E3 ubiquitin-protein ligase) pathway [48]. In vivo studies showed that Comp 5 significantly reduced tumor volume and weight in xenograft mouse models without significant toxicity [48]. These findings suggest that Comp 5, as a novel small-molecule activator of SIRT1, could be a promising candidate for glioblastoma therapy by inducing autophagic and mitophagic cell death [48].

Liu et al. indicated that urolithin A (UA) significantly inhibits the growth of glioblastoma cells (U251 and U118 MG) in a dose-dependent manner, reducing cell proliferation, migration, invasion, and colony formation [49]. UA treatment leads to cell cycle arrest, particularly at the G2/M phase, and increases apoptosis in glioblastoma cells [49]. Research indicates that UA increases the expression levels of SIRT1 and FOXO1 in glioblastoma cells, with SIRT1 playing a crucial role in mediating UA’s effects [49]. In animal models, UA treatment results in a significant reduction in tumor size and weight in nude mice implanted with glioblastoma cells [49]. The proposed mechanism of action is that UA exerts its effects by regulating the SIRT1-FOXO1 axis through the ERK (Extracellular Signal-Regulated Kinase) and AKT (protein kinase B) signaling pathways [49]. Given its ability to penetrate the blood–brain barrier and its demonstrated anti-cancer effects, UA is considered a promising candidate for glioblastoma treatment [49].

SIRT1 plays a significant role in glioma development, and its activity is regulated by SENP1 (sentrin-specific protease 1) [50]. Specifically, SIRT1 inhibits the activity of NF-κB through deacetylation, which helps suppress pathways that lead to tumor growth and malignancy in gliomas [50]. Downregulation of SIRT1 reverses the inhibitory effects of SENP1 depletion on the malignant phenotype of glioma cells, indicating that SIRT1 is crucial for maintaining the malignant characteristics of glioma cells [50]. The interplay between SIRT1 and NF-κB is complex, as downregulation of NF-κB can reverse the activating effects of SIRT1 on glioma cell malignancy [50]. This suggests that SIRT1’s inhibition of NF-κB is essential for controlling glioma cell behavior.

Wang et al. found that high concentrations of glucose can downregulate SIRT1 expression, leading to increased levels of acetylated HMGB1 (High-Mobility Group Box 1 protein), which is associated with promoting glioma malignancy [51]. Glucose influences the HMGB1 signaling pathway through SIRT1, where low SIRT1 levels due to high glucose result in the activation of HMGB1, promoting the proliferation, migration, and invasion of glioma cells while inhibiting apoptosis [51]. Silencing SIRT1 increases epithelial markers and decreases mesenchymal markers, suggesting that SIRT1 promotes EMT (epithelial–mesenchymal transition) in glioma cells, facilitating their invasive capabilities [51].

In gliomas, SIRT1 plays a crucial role in the conversion of microglia into tumor-supporting cells [52]. Glioma cells induce the nuclear localization of SIRT1 in microglia, which is essential for changes in histone acetylation [52]. Activated SIRT1 leads to the deacetylation of hMOF, reducing H4K16 acetylation, which promotes a tumor-supporting phenotype in the microglia [52]. Manipulating H4K16 acetylation levels, influenced by SIRT1 and hMOF, modulates the tumor-supporting functions of microglia [52]. Targeting the SIRT1-hMOF pathway represents a novel therapeutic strategy to inhibit the tumor-supporting actions of microglia in glioma, potentially improving patient outcomes [52].

In the study by Deng et al., miR-376a exhibits tumor-suppressing behavior in glioma cells, with its expression being lower in glioma cells compared with normal astrocytes [53]. Overexpression of miR-376a leads to the inhibition of SIRT1, YAP1 (Yes-associated protein 1), and VEGF (Vascular Endothelial Growth Factor) expression, consequently suppressing the proliferation, migration, and angiogenesis of glioma cell lines [53]. Mechanistically, miR-376a directly targets and inhibits SIRT1, as confirmed by luciferase assays [53]. SIRT1 overexpression upregulates YAP1 and VEGF, promoting glioma cell proliferation, migration, and angiogenesis [53]. In xenograft models, the ectopic expression of miR-376a results in lower tumor volumes and weights along with a slower growth curve [53]. This overexpression inhibits YAP1/VEGF signaling and angiogenesis by inhibiting SIRT1 in xenograft tissues [53]. These findings suggest that miR-376a’s downregulation of SIRT1 leads to decreased YAP1 and VEGF signaling, which in turn suppresses glioma cell proliferation, migration, and angiogenesis [53].

In glioma stem cells, SIRT1 acts as a pluripotent marker, exhibiting increased expression in stem media compared with normal media [54]. While SIRT1 inhibition via EX527 treatment does not directly alter cell proliferation, it reduces the expression of stemness markers like Sox-2 (SRY–Box Transcription Factor 2) and Oct-4 (octamer-binding transcription factor 4) and decreases the capacity to form gliomaspheres [54]. SIRT1 deacetylates p53 (tumor protein p53), rendering it inactive and initiating a cascade of events with oncogenic potential [54]. Given that cancer stem cells are responsible for drug resistance and tumor relapse, understanding the mechanism of SIRT1 action in these cells could pave the way for more target-specific therapies [54].

SIRT1 is a crucial factor for maintaining cancer stemness in neural stem cells and glioma stem cells, which is essential for their survival and tumorigenicity, particularly in a p53-dependent manner [55]. SIRT1 expression is significantly higher in cancerous neural stem cells compared with normal human neural stem cells [55]. Depletion of SIRT1 leads to a marked reduction in cell growth and an increase in apoptosis in these cancerous neural stem cells, but this effect is not observed in the U87 glioma cell line, suggesting a specific role in cancer cells with neural stemness [55]. The loss of SIRT1 results in increased levels of p53 and its acetylation, associated with the activation of apoptotic pathways [55]. This indicates that SIRT1 helps to suppress p53 activity, allowing cancer cells to evade cell death [55]. Targeting sirtuin 1 could be a promising therapeutic strategy for treating cancers characterized by stem-like properties, as inhibiting SIRT1 may restore p53 function and induce cell death in these cancer cells [55].

SIRT1 also plays a significant role in medulloblastoma, influencing cell growth and survival. Studies have shown SIRT1 expression in a substantial percentage of medulloblastoma tissues, suggesting its involvement in tumor development and progression [56]. Notably, SIRT1 expression is higher in aggressive subtypes of medulloblastoma (large cell/anaplastic—79.07% and classic medulloblastomas—60.29%) compared with less aggressive ones (nodular/desmoplastic subtype—22.22%), indicating a potential link between SIRT1 and tumor aggressiveness [56]. Inhibiting SIRT1 expression or activity leads to cell cycle arrest and increased apoptosis in medulloblastoma cells [56]. Therefore, targeting SIRT1 could be a promising therapeutic strategy for treating this brain tumor [56].

Resveratrol can reduce SIRT1 expression in medulloblastoma cells at both transcriptional and translational levels without affecting its enzymatic activity [57]. This suggests that while resveratrol inhibits SIRT1 expression, it does not alter its deacetylating functions [57]. The conclusion is that high levels of SIRT1 expression may promote the formation of medulloblastoma, and resveratrol could be a potential therapeutic agent for it by regulating SIRT1 expression without affecting its activity [57].

SIRT1 activation can be a selective strategy to target IDH-mutant tumors [58]. Activating sirtuin 1 significantly increases the depletion of nicotinamide adenine dinucleotide (NAD+) levels in IDH-mutant tumor cells, which rely heavily on NAD+ for survival [58]. Combining SIRT1 activation with NAMPT (Nicotinamide Phosphoribosyltransferase) inhibition markedly increases cytotoxicity [58]. Both genetic overexpression of SIRT1 and the use of pharmacological SIRT1-activating compounds inhibit the growth of IDH1-mutant tumor cells [58]. Relatively nontoxic STACs, alone or with NAMPT inhibitors, could alter the growth trajectory of IDH-mutant gliomas, minimizing the toxicity of chemotherapy [58].

## 3. SIRT2

SIRT2 (sirtuin 2) is characterized by a central catalytic domain flanked by flexible N and C termini, which are subject to alternative splicing and post-translational modifications such as phosphorylation [59]. The enzyme is primarily located in the cytoplasm, where it interacts with microtubules and other substrates, facilitating processes like tubulin deacetylation [60,61]. SIRT2’s activity is dependent on NAD+, and its structure allows it to remove acetyl and longer-chain acyl groups from lysine residues on target proteins [62,63].

SIRT2 is a versatile protein involved in numerous cellular processes [64]. It participates in the regulation of the cell cycle, gene expression, and chromatin dynamics, acting as a deacetylase for both histone and non-histone proteins, thereby influencing transcriptional silencing and protein stability [59,65]. SIRT2 also plays a role in metabolic pathways, affecting energy metabolism and genome stability, and it has been implicated in the pathogenesis of cancer and neurodegenerative diseases [62,64,66]. Furthermore, SIRT2 regulates microtubule dynamics and Golgi structure by modulating the acetylation levels of proteins like GRASP55 (Golgi reassembly-stacking protein of 55 kDa), which is crucial for post-mitotic Golgi assembly [67]. SIRT2 has also been described as both an oncogene and a tumor suppressor [68]. As a tumor suppressor, SIRT2 maintains genome stability and prevents oncogenic transformations by regulating the DNA damage response through deacetylating key components like ATRIP (ATR-interacting protein) and CDK9 (Cyclin-dependent kinase 9) [69,70]. This ensures proper replication stress recovery and genome integrity [69,70]. SIRT2 also modulates the mitotic deposition of H4K20 (lysine 20 on histone H4) methylation, which is critical for chromosomal stability and cell cycle regulation [69]. However, SIRT2 can also promote tumorigenesis by supporting cancer cell proliferation and survival. For example, in glioblastoma, SIRT2 deacetylates p73, a tumor suppressor, thereby inactivating its transcriptional activity and fostering tumor growth [71]. Similarly, SIRT2 inhibitors have shown promise in targeting ATRX (alpha-thalassemia mental retardation X-linked)-deficient gliomas, highlighting its role in driving oncogenic phenotypes in specific tumor subtypes [72].

SIRT2’s activity is modulated by phosphorylation at specific sites, which can enhance its deacetylation activity on certain substrates [59]. SIRT2 expression is also influenced by stress conditions, such as chronic stress, which can downregulate its expression and affect synaptic plasticity-related genes [73].

### SIRT2 in CNS Tumors

SIRT2 plays a significant role in glioblastoma by promoting the proliferation and tumorigenicity of glioblastoma cells, including glioblastoma stem cells [71]. It regulates the transcriptional activity of p73 (tumor protein p73), a tumor suppressor, by deacetylating its C-terminal lysine residues, leading to p73 inactivation [71]. This inactivation is critical for the growth and survival of glioblastoma cells [71]. Thus, the SIRT2-mediated inactivation of p73 is a crucial mechanism in the tumorigenicity of glioblastoma, suggesting that targeting SIRT2 could be a promising therapeutic strategy [71]. By inhibiting SIRT2, it may be possible to restore the function of p73, potentially reducing the tumorigenicity of glioblastoma cells [71].

The study by Imaoka et al. focused on the expression and localization of the SIRT2 protein in human gliomas, specifically, glioblastoma and diffuse astrocytoma [74]. Using immunohistochemistry to analyze samples from 23 glioblastoma patients, 8 diffuse astrocytoma patients, and 5 healthy individuals, it established a SIRT2 labeling index (SIRT2-LI) to measure the percentage of cells with SIRT2 localized in the nucleus [74]. The results showed that the mean SIRT2-LI was significantly higher in glioblastoma samples compared with diffuse astrocytoma samples and normal controls [74]. The study found a positive correlation between SIRT2-LI and the malignancy of gliomas [74]. Patients with low SIRT2-LI had a significantly longer survival time than those with high SIRT2-LI, suggesting that higher levels of nuclear SIRT2 expression are associated with worse survival outcomes [74]. The study concluded that SIRT2 expression and its localization could be indicative of glioma malignancy and may help predict patient survival, highlighting the potential of SIRT2 as a target for therapeutic strategies in treating glioblastoma [74].

Malgulwar et al. studied ATRX-deficient malignant gliomas characterized by mutations in the ATRX gene, which lead to changes in chromatin structure and gene expression, contributing to cancer progression [72]. Researchers identified sirtuin 2 as a key player in the oncogenic features of these gliomas [72]. They found that SIRT2 is overexpressed in these tumors and correlates with poor patient outcomes [72]. Inhibiting SIRT2 with small-molecule inhibitors could reverse the transcriptional changes caused by ATRX deficiency, leading to increased levels of acetylated histones [72]. The results showed that SIRT2 inhibition significantly reduced the motility of ATRX-deficient cells and promoted cellular senescence [72]. In vivo studies demonstrated that SIRT2 inhibitors slowed down the growth of ATRX-deficient glioma xenografts and also induced senescence in these tumors, suggesting a potential therapeutic strategy for treating this aggressive cancer type [72].

Le et al. found that SIRT2 is underexpressed in human glioma tissues and cell lines [50]. Overexpression of sirtuin 2 decreases cell proliferation and colony-formation capacity and induces cellular apoptosis by upregulating cleaved caspase 3 and BAX while downregulating the anti-apoptotic protein Bcl-2 (B-cell lymphoma 2 protein) [50]. Conversely, SIRT2 knockdown yields opposing results [50]. SIRT2 overexpression inhibits miR-21 expression and is insufficient to reduce cell proliferation and colony formation or induce apoptosis when miR-21 is knocked down in glioma cells [50]. Mechanistically, SIRT2 deacetylates p65 at K310 and blocks p65 binding to the promoter region of miR-21, thus regressing the transcription of miR-21 [50]. Sirtuin 2 may act as a tumor suppressor gene in human gliomas and be critical in human glioma via the NF-κB–miR-21 pathway [50].

SIRT2 activity is essential for the survival of glioma cells, and its reduction leads to both necrosis and caspase-3-dependent apoptosis of C6 glioma cells [75]. A study by He et al. using flow cytometry-based Annexin V assays and caspase-3 immunostaining confirmed that decreased SIRT2 activity induces apoptosis in C6 glioma cells via the caspase-3-dependent pathway [75]. Experiments with SIRT2 siRNA also demonstrate that decreased SIRT2 leads to both necrosis and apoptotic changes in C6 glioma cells [75]. These findings suggest that inhibiting SIRT2 might be a novel therapeutic strategy for gliomas [75].

A study by Sayd et al. highlights the significant role of SIRT2 in glioblastoma stem cells (GSCs) and its interaction with resveratrol [76]. SIRT2 is specifically expressed in GSCs but not in normal neural stem cells, suggesting a unique function in cancer stem cell biology [76]. SIRT2 was identified as a key mediator of the effects of resveratrol on GSCs [76]. When researchers inhibited SIRT2 activity, the inhibitory effect of resveratrol on GSC proliferation was significantly reduced, indicating SIRT2’s involvement in how resveratrol affects the growth of these cancer cells [76]. Resveratrol causes GSCs to stop progressing through the cell cycle, and this cell cycle arrest was linked to SIRT2 activity [76]. When SIRT2 was inhibited, the ability of resveratrol to halt the cell cycle was diminished, suggesting that SIRT2 is crucial for this specific action of resveratrol [76]. While SIRT2 was important for the proliferation effects of resveratrol, the necrosis induced by high doses of resveratrol was found to be independent of SIRT2 activity [76]. This highlights that SIRT2’s role is more about regulating cell growth rather than cell death in this context [76].

The study by Alimova et al. demonstrates that SIRT2 inhibition significantly impacts ATRT (atypical teratoid/rhabdoid tumors) cell growth and survival [77]. Inhibiting SIRT2, either through shRNA or the compound Thiomyristoyl, significantly decreased the growth of ATRT cells [77]. This inhibition extends to the clonogenic potential of ATRT cells, indicating a role in their ability to form colonies, a key characteristic of cancer cell aggressiveness [77]. SIRT2 inhibition also leads to cell cycle arrest in ATRT cells and suppresses genomic programs associated with pluripotency [77]. This results in a significant reduction in stem cell frequency and decreased tumor-sphere formation, indicating a shift away from stem-like properties in ATRT cells [77]. In animal models, SIRT2 inhibition resulted in decreased oncogenic markers and an increase in neuronal differentiation markers, suggesting that targeting SIRT2 may promote differentiation in ATRT cells, potentially leading to less aggressive tumor behavior [77]. Furthermore, SIRT2 inhibition induces apoptosis in ATRT cells and, in orthotopic xenograft models, decreases tumor growth and prolongs survival [77]. Collectively, these findings highlight SIRT2 as a critical molecular target in SMARCB1-deleted ATRT, indicating that therapeutic strategies aimed at SIRT2 inhibition may offer a novel approach for treating this highly aggressive pediatric brain tumor.

## 4. SIRT3

SIRT3 is a NAD+-dependent deacetylase that is primarily located in the mitochondria [78,79]. It regulates the acetylation of mitochondrial proteins, influencing mitochondrial metabolism and homeostasis [78,79,80,81].

SIRT3 is involved in several critical biological processes, including oxidative stress management, inflammation, DNA damage repair, and apoptosis [79,82,83]. These functions are essential for preserving cellular equilibrium and offering a defense against diverse pathologies [79,82]. SIRT3 exerts a considerable influence on mitochondrial metabolic processes, encompassing the tricarboxylic acid cycle, ATP synthesis, and the management of reactive oxygen species (ROS) [36,78,82,84]. This regulatory function is vital for maintaining energy homeostasis and ensuring cellular viability [78,82]. SIRT3 also influences autophagy, a process necessary for cellular maintenance and the recycling of cellular components, which is important in preventing diseases such as cancer (although in this case, it may act as a promoter or a suppressor) and neurodegenerative disorders [85,86].

SIRT3 expression is subject to regulation by diverse elements, encompassing nutritional state, physical activity, and ambient temperature. These lifestyle determinants can modulate SIRT3 functionality, thereby impacting mitochondrial performance and comprehensive cellular health [84].

### SIRT3 in CNS Tumors

SIRT3 expression in skeletal muscle is influenced by nutritional states such as feeding, fasting, and caloric restriction, which impacts both mitochondrial oxidation and insulin signaling [87]. This regulatory mechanism is particularly significant in the context of metabolic disorders like diabetes [87]. Small molecules, including those derived from natural sources, can modulate SIRT3’s activity. These activators present potential therapeutic avenues for addressing diseases linked to mitochondrial dysfunction [81,88].

SIRT3 is identified as a crucial enzyme in glioblastoma cells, regulating mitochondrial metabolism by modulating the acetylation of lysine residues on mitochondrial enzymes [89]. Inhibiting SIRT3 leads to notable changes in the protein synthesis machinery, highlighting its vital role in maintaining the metabolic characteristics of these cells [89]. Lysine acetylation significantly influences the metabolic phenotype of glioblastoma cells, and SIRT3 is essential for regulating the balance of metabolic processes in these cancer cells [89]. This leads to potential novel targets for future investigations and emphasizes the critical function of SIRT3 in mitochondrial metabolism and its broader implications for cellular energetics in glioblastoma [89].

Le et al. found that SIRT3 expression is significantly higher in GBM (glioblastoma) tissues compared with normal brain tissues, suggesting its involvement in GBM progression [90]. Interestingly, SIRT3 protein levels increase during RAS-selective lethal 3-induced ferroptosis in GBM cells, indicating a potential role in the cellular response to ferroptosis [90]. Inhibiting SIRT3 expression and activity in GBM cells increases their sensitivity to RSL3-induced ferroptosis both in vitro and in vivo, suggesting that targeting SIRT3 could enhance ferroptosis in GBM [90]. Mechanistically, SIRT3 inhibition leads to the accumulation of ferrous iron and reactive oxygen species in mitochondria, triggering mitophagy, which is essential for maintaining mitochondrial health [90]. RNA-sequencing analysis has revealed that SIRT3 knockdown upregulates the mitophagy pathway and downregulates SLC7A11 (Solute carrier family 7 member 11), a protein crucial for glutathione synthesis, which is vital for cellular defense against oxidative stress [90]. Forced expression of SLC7A11 in GBM cells with SIRT3 knockdown can restore cystine uptake and GSH (glutathione) levels, partially rescuing cell viability during RSL3 treatment, highlighting SIRT3’s protective role against ferroptosis by regulating SLC7A11 [90]. Overall, SIRT3 protects GBM cells from ferroptosis through mechanisms involving mitophagy and the regulation of SLC7A11, making it a promising therapeutic target in combination with ferroptosis induction for treating glioblastoma [90].

In their study, Shi et al. found that fraxinellone downregulates SIRT3 expression in GBM cells, suggesting SIRT3’s significant role in GBM progression, as its reduction inhibits cell proliferation and migration [91]. Overexpressing SIRT3 partially restores the inhibition of cell proliferation and migration in fraxinellone-treated GBM cells, indicating a protective role against fraxinellone’s effects and highlighting its importance in GBM progression signaling pathways [91]. Fraxinellone treatment increases ROS (reactive oxygen species) levels in GBM cells, likely linked to SIRT3 downregulation, contributing to the apoptosis of GBM cells induced by fraxinellone [91]. In vivo, fraxinellone inhibits GBM tumor growth through inactivation of the SIRT3 signaling pathway, reinforcing SIRT3’s critical role in GBM tumorigenesis and its potential as a therapeutic target [91].

Qiao et al. explored the essential role of SIRT3, a mitochondrial deacetylase, in regulating mitophagy and apoptosis in cancer cells, particularly under hypoxic conditions [92]. Sirtuin 3 was shown to positively regulate autophagy, as its overexpression increased LC3-II levels, while its silencing reduced LC3-II and Atg5-12 complex levels and elevated p62, indicating suppressed autophagic activity [92]. In hypoxic glioma cells, SIRT3 promoted mitophagy, evidenced by the co-localization of GFP-LC3 with mitochondria and the reduction of mitochondrial mass—effects diminished when Sirt3 was inhibited [92]. Mechanistically, SIRT3 enhanced the interaction between VDAC1 and Parkin, a critical step in mitophagy, without altering hexokinase II expression but influencing its subcellular localization [92]. Suppressing SIRT3 heightened mitochondrial damage, as shown by greater loss of membrane potential and increased ROS levels under hypoxia, and sensitized glioma cells to hypoxia-induced apoptosis [92]. Additionally, sirtuin 3 knockdown promoted the ROS-dependent proteasomal degradation of anti-apoptotic proteins Mcl-1 and survivin, significantly reducing their stability—an effect reversible by proteasome or ROS inhibitors [92]. These findings underscore SIRT3’s protective role in maintaining mitochondrial function and preventing apoptosis in hypoxic tumor environments [92].

In glioblastoma cells, SIRT3 expression is significantly induced after radiation exposure, indicating an adaptive response to therapy [93]. This induction is linked to the activation of the SIRT3 promoter by the transcription factor NF-kB, leading to increased SIRT3 levels that support mitochondrial function and energy production [93]. The phosphorylation of SIRT3 at Thr150 and Ser159 by cyclin B1-CDK1 is enhanced post-radiation, which is crucial for SIRT3’s activity in managing energy needs and resisting radiation damage [93]. Glioblastoma cells expressing a mutant SIRT3 lacking phosphorylation sites show reduced mitochondrial function and increased radiation sensitivity [93]. These findings suggest SIRT3 plays a significant role in glioblastoma cell radioresistance, highlighting its potential as a therapeutic target to improve radiation treatment effectiveness [93].

In the study by Haq et al., SIRT3 was significantly upregulated in glioma patients compared with healthy controls, with a *p*-value of 0.0322 indicating statistical significance [94]. This upregulation suggests a potential link to tumor aggressiveness, where higher levels of SIRT3 may be associated with an increased survival capacity of tumor cells [94]. Unlike SIRT4, which is downregulated in glioma patients, SIRT3’s upregulation indicates that different sirtuins may have distinct roles in glioma progression [94]. ROC (Receiver-operating characteristic) curve analysis suggests that SIRT3 has good diagnostic sensitivity in glioma patients, suggesting it could be a valuable biomarker for diagnosis and prognosis [94].

Luo et al. found that SIRT3 levels are lower in glioma tissues compared with normal brain tissues, indicating a potential link between SIRT3 expression and tumor behavior [95]. However, higher levels of SIRT3 are significantly associated with advanced tumor grades and shorter overall survival times in glioma patients, suggesting SIRT3 as an independent prognostic factor [95]. In laboratory experiments, SIRT3 overexpression increased cell viability, while silencing SIRT3 reduced cell growth, indicating SIRT3 promotes glioma cell survival [95]. SIRT3 interacts with Ku70, enhancing its ability to prevent BAX from entering the mitochondria, thus reducing cell death [95]. The conclusion drawn from the study is that SIRT3 not only serves as a potential biomarker for glioma prognosis but also plays a significant role in glioma progression through its interaction with Ku70.

Cheng et al. found that linalool significantly reduces both the mRNA and protein expression levels of SIRT3 in U87-MG glioma cells, suggesting it may inhibit SIRT3 activity [96]. Linalool treatment decreases the interaction between SIRT3 and SOD2 (mitochondrial superoxide dismutase), potentially leading to increased oxidative stress [96]. Overexpression of SIRT3 inhibits the increase in mitochondrial ROS levels induced by linalool, indicating a protective role against oxidative stress [96]. Furthermore, SIRT3 overexpression inhibits linalool-induced apoptotic cell death and the decrease in cell viability, highlighting its importance in maintaining cell survival, suggesting SIRT3 could be a potential therapeutic target against glioma [96]. Overall, linalool’s inhibitory effects on glioma cell viability are linked to the downregulation of SIRT3, which in turn affects SOD2 activity and increases ROS levels [96].

In their study, Xing et al. proved that SIRT3 is significantly enriched in CD133+ glioblastoma stem cells, indicating its potential role in maintaining stemness in these cells [97]. Sirtuin 3 was shown to promote mitochondrial respiration and reduce oxidative stress, which is crucial for maintaining GSCs stemness, and to epigenetically regulate CD133 expression through succinate [97]. During GSCs’ differentiation into bulk tumor cells, SIRT3 is degraded through the autophagy–lysosome pathway, triggered by glutamine deprivation, which restricts CD133 expression and disrupts GSC stemness [97]. Targeting glutamine metabolism to induce the autophagic degradation of SIRT3 could be a novel strategy to eliminate GSCs and overcome glioblastoma resistance to therapy [97].

Overexpression of SIRT3 in glioma stem cells has been also found by Park et al. [98]. Sirtuin 3 cooperates with TRAP1 to enhance mitochondrial respiration without increasing ROS production, which is vital for GSCs’ survival and proliferation; inhibiting either SIRT3 or TRAP1 (Tumor Necrosis Factor Receptor-Associated Protein 1) leads to metabolic dysregulation, increased ROS, and loss of stemness [98]. SIRT3 stabilizes TRAP1 and enhances its chaperone activity, which is necessary for the proper functioning of mitochondrial electron transport chain complexes and crucial for maintaining high mitochondrial respiration rates in GSCs, helping them adapt to low-nutrient conditions [98]. Knocking down SIRT3 increases mitochondrial ROS production, indicating its protective role against oxidative stress in GSCs, linked to the deacetylation of proteins like SOD2 (superoxide dismutase 2), involved in ROS metabolism [98]. Targeting the SIRT3–TRAP1 interaction could be a promising therapeutic strategy by disrupting this interplay to reduce the stemness and tumorigenic potential of GSCs [98].

## 5. SIRT4

SIRT4, a member of the sirtuin family of NAD+-dependent enzymes, plays crucial roles in metabolism, stress response, and aging. Primarily localized in the mitochondria, SIRT4 regulates mitochondrial functions and energy metabolism [99,100,101]. Structurally, it shares a conserved catalytic core with other sirtuins, but it possesses unique enzymatic activities, including ADP-ribosyltransferase, deacetylase, lipoamidase, and long-chain deacylase functions [100]. These properties allow SIRT4 to modulate substrate proteins through post-translational modifications like ADP ribosylation, delipoylation, deacetylation, and long-chain deacylation [100]. In addition to its mitochondrial localization, SIRT4 dynamically localizes at centrosomes during the cell cycle, peaking in the G2 phase and early mitosis, suggesting roles in cell cycle regulation and microtubule dynamics [99].

SIRT4 is a key regulator of the cellular metabolism, particularly in fatty acid oxidation, glutamine metabolism, and insulin secretion. In human skeletal muscle cells, SIRT4 knockdown increases mitochondrial capacity to oxidize fatty acids, suggesting its role in modulating substrate utilization [102]. Additionally, SIRT4 inhibits glutamine metabolism by promoting the ADP ribosylation of glutamate dehydrogenase 1, thereby reducing glutamine-dependent ATP production [100,103,104]. In liver fibrosis, SIRT4 enhances the cytotoxicity of natural killer cells toward activated hepatic stellate cells by activating the AMPKα/P-p53/NKG2DL pathway, thereby reversing fibrosis [105]. Sirtuin 4 promotes neuronal apoptosis via the STAT2-SIRT4-mTOR pathway, suggesting its role in neurodegeneration [106]. Its localization at centrosomes and interaction with microtubule components indicate its involvement in mitotic progression and cell proliferation [99]. SIRT4 exhibits tumor-suppressive functions in various cancers, such as prostate cancer, where it inhibits cell progression by modulating p21 nuclear translocation and glutamine metabolism [104]. In colorectal cancer, SIRT4 suppresses malignant phenotypes by activating glutaminase and inhibiting the AKT/GSK3β/CyclinD1 pathway [103]. Similarly, in neuroblastoma, sirtuin 4 reduces tumor cell proliferation and mitochondrial energy production, suggesting its potential as a therapeutic target [107,108].

SIRT4 expression is tightly regulated at both the transcriptional and post-translational levels. Transcriptional regulation involves factors like E2F1 (E2F transcription factor 1), CEBPβ (CCAAT/enhancer-binding protein beta), and HOXA5 (Homeobox protein A5), which transcriptionally activate SIRT4 expression, while IRF4 (Interferon Regulatory Factor 4), PAX4 (Paired box 4), and CREB1 (cAMP response element binding protein 1) act as repressors in bovine adipocytes [109]. These transcription factors bind to specific regions in the SIRT4 promoter, modulating its expression during adipocyte differentiation [109]. Post-translational regulation influences SIRT4 activity through interactions with mitochondrial and cytoskeletal proteins, suggesting a role in regulating microtubule dynamics and mitotic progression [99]. SIRT4 expression is often dysregulated in disease states; for example, it is upregulated in Alzheimer’s disease, promoting neuronal apoptosis, while decreased expression is observed in colorectal cancer, highlighting its tumor-suppressive role [100,106]. In liver fibrosis, SIRT4 expression is downregulated, impairing NKs’ (natural killer cells) cell cytotoxicity against HSCs (Hematopoietic Stem Cells) [105].

### SIRT4 in CNS Tumors

Akkulak et al. investigated SIRT4 expression in glioblastoma (U87) cells and intracranial tumors, finding lower SIRT4 mRNA expression across all tumor types, though not statistically significant [110]. However, SIRT4 protein expression was elevated in the U87 glioblastoma cell line compared with immortalized human astrocytes [110]. Alongside SIRT4, the study examined glutamate dehydrogenase and glutamine synthetase, noting elevated SIRT4 and GS protein expressions in U87 cells, while GDH protein expression was reduced, suggesting a complex interplay in glutamate metabolism and excitotoxicity in tumors [110].

SIRT4 overexpression in the A172 glioma cell line significantly increases cell viability after treatment with kainic acid, suggesting a protective role against excitotoxicity in glioma cells [111]. Following SIRT4 overexpression and kainic acid treatment, a reduction in glutamate levels is observed, indicating that SIRT4 helps in managing glutamate metabolism, thereby preventing excessive glutamate accumulation [111]. SIRT4 overexpression leads to increased levels of GLT-1 (glutamate transporter-1) and glutamate dehydrogenase, enhancing the cell’s ability to absorb excess glutamate and supporting the metabolism of glutamate [111]. Interestingly, SIRT4 overexpression results in decreased levels of glutamine synthetase, preventing the formation of glutamine and reducing the potential for glutamate accumulation [111]. These findings suggest that SIRT4 has a protective effect against excitotoxicity by modulating glutamate metabolism, highlighting the potential of targeting SIRT4-related pathways for developing therapeutics aimed at preventing excitotoxicity and related cell death [111].

According to Xuan et al., SIRT4 demonstrates significant downregulation in glioma patients compared with healthy controls, suggesting its potential as a tumor suppressor in gliomas [94]. This downregulation implies that reduced SIRT4 expression could contribute to tumor development [94]. The downregulation of SIRT4 in glioma highlights its potential as a biomarker for diagnosis and prognosis in glioma patients [94].

Wang et al. found that SIRT4 gene expression was significantly lower in neuroblastoma (NB) tumor tissues compared with adjacent normal tissues [107]. Specifically, the expression level was measured to be (1.03 ± 0.23) in tumor tissues versus (1.45 ± 0.13) in normal tissues, indicating a clear downregulation of SIRT4 in cancerous cells [107]. There was a notable correlation between low SIRT4 expression and advanced stages of neuroblastoma as well as lymph node metastasis [107]. Patients with higher International Neuroblastoma Staging System stages had a higher proportion of low SIRT4 expression, suggesting that SIRT4 may play a role in tumor progression [107]. The study revealed that patients with low SIRT4 expression had significantly shorter survival times compared with those with high expression [107]. This suggests that SIRT4 could be an important prognostic factor in neuroblastoma, with lower levels indicating a worse prognosis [107]. Overexpressing SIRT4 in human NB cell lines led to a significant reduction in cell proliferation, invasion, and migration, indicating that SIRT4 functions as a tumor suppressor [107]. The study also found that SIRT4 overexpression reduced mitochondrial respiration and energy production in NB cells [107]. Overall, the findings indicate that SIRT4 plays a critical role in neuroblastoma by acting as a tumor suppressor [107].

## 6. SIRT5

SIRT5 is a mitochondrial enzyme belonging to the class III NAD+-dependent deacetylases, characterized by its ability to remove negatively charged acyl groups from lysine residues [112,113]. Structurally, SIRT5 is composed of eight exons and exists in two isoforms, encoding proteins of 310 and 299 amino acids, respectively [114]. The enzyme’s active site is specifically adapted to recognize and catalyze the removal of succinyl, malonyl, and glutaryl groups, with structural studies highlighting the importance of conserved hydrogen bonds for substrate recognition [115].

SIRT5 plays a crucial role in regulating various metabolic pathways, including glycolysis, the tricarboxylic acid cycle, fatty acid oxidation, and the electron transport chain [116]. It is involved in the detoxification of reactive oxygen species and the regulation of myocardial energy metabolism, contributing to heart physiology and stress responses [116,117]. In cancer, SIRT5 exhibits a dual role, acting as a tumor promoter or suppressor depending on the context. SIRT5 has been shown to contribute to cisplatin resistance in ovarian cancer through modulation of the Nrf2/HO-1 pathway and to suppress tumor growth in gliomas by regulating mitochondrial metabolism [8]. Furthermore, its involvement in synaptic remodeling and neuroplasticity suggests a potential role in neurodegenerative diseases [8].

The SIRT5 gene, located on chromosome 6p23, is predominantly expressed in heart muscle cells and lymphoblasts [114]. Its expression is modulated during various physiological and pathological conditions, such as increased expression in diabetic cardiomyopathy, where it ameliorates cardiac lipotoxicity by enhancing fatty acid metabolism [117]. SIRT5’s activity is regulated through its interaction with specific substrates and inhibitors, with ongoing research focusing on developing potent and selective inhibitors for therapeutic applications [112,118].

### SIRT5 in CNS Tumors

Sirtuin 5 plays a crucial role in regulating cellular metabolism, which is often disrupted in cancer cells, leading to increased tumor growth, especially in glioblastoma [8]. Higher levels of SIRT5 expression are linked to a more favorable prognosis for glioma patients, suggesting a protective effect against tumor progression [8]. Experimental results show that knocking down SIRT5 significantly enhances the growth of glioblastoma cells, implying that SIRT5 functions as a tumor suppressor [8]. This regulation is critical because altered metabolism is a hallmark of cancer, and SIRT5’s role in this process may contribute to its tumor-suppressive effects [8]. SIRT5 is also found to be significantly correlated with pathways involved in synaptic remodeling, indicating that SIRT5’s influence extends beyond metabolism, potentially affecting neuroplasticity and the brain’s structural adaptations in response to glioma [8].

The study by Chen et al. focused on the role of SIRT5 in glioblastoma [119]. The researchers found that SIRT5 expression was significantly decreased in GBM tissues compared with normal tissues, indicating its potential role in tumor suppression [119]. A comprehensive analysis revealed that lower levels of SIRT5 were associated with shorter survival times for patients with GBM [119]. This suggests that SIRT5 could serve as a prognostic biomarker, meaning it may help predict patient outcomes. The researchers also discovered a negative correlation between SIRT5 expression and DNA methylation status, meaning that as DNA methylation increases, SIRT5 expression tends to decrease [119]. Functional enrichment analysis showed that SIRT5 is potentially involved in important biological processes, such as epithelial–mesenchymal transition and cell communication [119]. In glioma patients, SIRT5 is significantly downregulated compared with healthy controls, suggesting it may act as a tumor suppressor in gliomas [119].

In glioma patients, SIRT5 is significantly downregulated compared with healthy controls, with a highly significant *p*-value of less than 0.0001 [94]. This downregulation suggests that SIRT5 may act as a tumor suppressor in gliomas [94]. Low SIRT5 levels could lead to increased levels of ROS and other metabolic disturbances, potentially promoting cancer progression [94]. Unlike SIRT3, which is upregulated in glioma patients, SIRT5’s downregulation highlights the different roles that these sirtuins play in cancer biology [94]. This difference suggests that while SIRT3 may contribute to tumor aggressiveness, SIRT5 may help protect against tumor development [94].

## 7. SIRT6

SIRT6, predominantly located in the nucleus and associated with chromatin, possesses a catalytic domain that interacts with nucleosomes, prying DNA from the nucleosomal entry–exit site and exposing the histone H3 N-terminal helix [120,121,122]. The zinc-binding domain of SIRT6 binds to the histone acidic patch using an arginine anchor, facilitating its deacetylation activity [122]. Additionally, SIRT6 has a Rossmann fold domain that binds to the DNA terminus, placing the NAD+ binding pocket close to the DNA exit site, which is crucial for its enzymatic activity [120].

SIRT6 is a multitasking enzyme involved in various crucial biological functions, including gene silencing, metabolism, DNA repair, antioxidant defense, inflammation, aging, and longevity [121,123,124,125,126,127,128]. It plays a role in histone modification, particularly in the deacetylation of histone H3, which is crucial for chromatin silencing and transcriptional regulation [129,130]. SIRT6 regulates energy metabolism by suppressing glycolysis, affecting photoreceptor cell survival, and protecting retinal ganglion cells from oxidative stress [123]. It impacts cellular homeostasis by regulating DNA repair, telomere maintenance, and glucose and lipid metabolism [124]. SIRT6’s enzymatic characteristics form the foundation of its ability to regulate various physiological and pathological processes [121].

SIRT6 expression is intricately regulated to maintain organism homeostasis, involving various upstream factors and interactions with multiple downstream substrates [131]. It influences immunosenescence, immunometabolism, and tumor immunology by regulating immune cells [121]. SIRT6 also modulates histone acetylation states, influencing chromatin structure and gene expression, particularly in cancer biology [129,132]. Inactivation of sirtuin 6 in cancer cells leads to the accumulation of nuclear ACLY protein, increasing nuclear acetyl-CoA pools and driving locus-specific histone acetylation [129]. Furthermore, SIRT6 may localize to SGs in the cytoplasm in response to stress and aid in recovery from stress that may arise from oxidative damage, heat shock, or deprivation of nutrients [133]. Exogenous substances like resveratrol, sirtinol, flavonoids, cyanidin, and quercetin can also affect the expression level of SIRT6 [128].

### SIRT6 in CNS Tumors

SIRT6 plays a crucial role in counteracting the effects of TRF1 knockdown in glioblastoma multiforme cells [134]. When TRF1 is inhibited, SIRT6 helps to reverse the decrease in cell viability, suggesting it has a protective role in GBM cell growth and proliferation [134]. Knocking down TRF1 leads to a decrease in SIRT6 expression through the P53 pathway, indicating that TRF1 may regulate SIRT6 levels, and when TRF1 is inhibited, it negatively affects SIRT6, which could contribute to cellular aging and autophagy [134].

Increasing SIRT6 levels in glioma cells, specifically the U87-MG and T98G cell lines, significantly reduces cell growth, decreases cell viability, and increases cell injury [135]. This overexpression induces apoptosis, as evidenced by a higher number of apoptotic cells in the T98G glioma cell line [135]. SIRT6 overexpression also leads to the translocation of apoptosis-inducing factor from the mitochondria to the nucleus, a critical step in the apoptosis process [135]. Furthermore, SIRT6 overexpression lowers oxidative stress levels in glioma cells by reducing reactive oxygen species and malondialdehyde levels and increasing antioxidant enzyme activity [135]. Importantly, SIRT6 overexpression inhibits the activation of the JAK2/STAT3 (Janus kinase 2/signal transducer and activator of transcription 3) signaling pathway, which is often overactive in gliomas, contributing to the anti-cancer effects of SIRT6 [135]. The study also found that SIRT6 levels were significantly lower in human glioblastoma multiforme tissues compared with adjacent non-tumor tissues, suggesting its role as a tumor suppressor [135].

According to Chen et al., SIRT6 is significantly downregulated in glioma tissues and cell lines compared with normal brain tissues, suggesting its association with glioma development [45]. Overexpression of SIRT6 in glioma cell lines (U87 and U251) decreases cell proliferation, migration, and invasion, indicating its role in suppressing the aggressive characteristics of glioma cells [45]. Conversely, knocking down SIRT6 increases cell proliferation, migration, and invasion in the same glioma cell lines, further supporting its function as a tumor suppressor [45]. SIRT6 overexpression decreases NOTCH3 (Neurogenic locus notch homolog protein 3) levels in U87 cells, suggesting that SIRT6 may inhibit NOTCH3 expression, reducing its tumor-promoting effects [45]. Furthermore, SIRT6 can counteract the aggressive behavior induced by NOTCH3 [45]. Overall, SIRT6 inhibits glioma cell proliferation, migration, and invasion through the negative regulation of NOTCH3 [45].

In glioma cells, SIRT6 is a direct target of miR-33a; when miR-33a is overexpressed, SIRT6 levels decrease at both the mRNA and protein levels, indicating that miR-33a negatively regulates SIRT6 [136]. SIRT6 expression is underexpressed in glioma tissues compared with normal tissues, and there is an inverse correlation between miR-33a and SIRT6 levels [136]. Restoring SIRT6 in glioma cells leads to increased levels of reactive oxygen species and lactate dehydrogenase, enhancing the sensitivity of glioma cells to oxidative stress-induced apoptosis [136]. Sirtuin 6 induces apoptosis by altering the expression of key proteins involved in the apoptotic pathway, such as increasing BAX and cleaved caspase-8 while decreasing Bcl-2 [136]. SIRT6 also inhibits the JAK2/STAT3 signaling pathway, which promotes cell survival and proliferation [136]. Dysregulation of the miR-33a/SIRT6 pathway contributes to glioma progression by promoting tumor growth and resistance to apoptosis; thus, targeting this pathway could provide new therapeutic strategies for treating glioma [137].

## 8. SIRT7

SIRT7, like other sirtuins, is characterized by a highly conserved catalytic domain, with key residues such as D118, Y119, and R120 being crucial for its function [138]. The three-dimensional structure of SIRT7 reveals a classic sirtuin catalytic region, while the N- and C-terminal regions vary among different species, indicating evolutionary adaptations [138]. SIRT7 primarily resides in the nucleolus, a nuclear compartment involved in ribosomal biogenesis and cellular stress responses [139].

SIRT7 is involved in maintaining genome stability by participating in DNA repair processes and regulating chromatin structure [140]. It recruits repair factors to sites of DNA damage, playing a critical role in the DNA damage response [140]. Additionally, SIRT7 regulates ribosome biogenesis and is involved in metabolic processes, including glucose and lipid metabolism [141]. It modulates target proteins in adipose and liver tissues, impacting metabolic diseases like type 2 diabetes and obesity [141]. In various cancers, including lung cancer, SIRT7 acts as an oncogene by destabilizing tumor suppressors, leading to the increased expression of pro-tumorigenic genes [142]. It is also linked to cell proliferation and oncogenic activity through its role in ribosome biogenesis and cell cycle regulation [143]. Furthermore, SIRT7 contributes to cellular stress resistance and aging processes; its ablation leads to genomic instability, premature aging, and metabolic dysfunctions [139,140].

SIRT7 expression is regulated by various factors, including its interaction with RNA, which enhances its catalytic efficiency [144]. RNA binding increases SIRT7’s ability to remove long-chain fatty acyl groups, highlighting its role in metabolic regulation [144]. The expression of SIRT7 is linked to cellular proliferation and is upregulated in conditions like cancer, while it is reduced in cardiovascular and bone diseases [138,145]. SIRT7’s activity is modulated by its localization within the cell, primarily in the nucleolus, where it controls nucleolar functions and ensures cellular integrity [139].

### SIRT7 in CNS Tumors

The study by Mu et al. indicates that sirtuin 7 is highly expressed in human glioma tissues, especially in higher-grade tumors, suggesting its significant role in glioma progression [146]. Analysis of patient samples revealed a positive correlation between SIRT7 levels and glioma malignancy, with higher expression in grade IV gliomas compared with grade II gliomas, indicating its potential as a prognostic marker [146]. Experiments using siRNA to reduce SIRT7 expression in glioma cell lines resulted in decreased cell proliferation and invasion, suggesting that SIRT7 promotes glioma cell growth and spread [146]. Further investigations showed that the downregulation of SIRT7 led to reduced activation of key signaling pathways, specifically the ERK and STAT3 pathways [146]. The conclusion drawn from the study is that SIRT7 could be a valuable target for glioma treatment, as inhibiting sirtuin 7 may reduce glioma cell proliferation and invasion, potentially improving patient outcomes [146].

Wang et al. found that SIRT7 expression is significantly upregulated in glioma tissues and cells, correlating positively with the pathological grade of glioma patients [147]. That suggests that higher SIRT7 levels may be associated with more aggressive tumor characteristics and poorer patient survival outcomes [147]. Both the knockdown and overexpression of SIRT7 affect glioma cell proliferation, apoptosis, and cell cycle progression [147]. Reducing SIRT7 levels enhances the cytotoxic effects of temozolomide (TMZ), indicating SIRT7’s role in mediating resistance to this treatment. MiR-148a-3p, a regulatory microRNA, targets sirtuin 7, and its expression is significantly decreased in glioma tissues and cells, suggesting its role in regulating SIRT7 levels and influencing glioma progression and therapy response [147]. In mouse xenotransplantation models, SIRT7 knockdown inhibits tumor growth and enhances the antitumor effects of TMZ, suggesting that targeting sirtuin 7 could be a promising therapeutic strategy to improve chemotherapy efficacy in glioma patients [147].

SIRT7 influences glioma progression by regulating IDH1 expression and metabolic pathways [148]. In glioma cells, SIRT7 knockdown leads to decreased IDH1 protein and mRNA levels, suggesting a positive regulatory role of SIRT7 on IDH1 expression [148]. This regulation is crucial for maintaining cellular levels of α-ketoglutarate (α-KG), impacting lipogenesis and gluconeogenesis in glioma cells [148]. SIRT7 regulates IDH1 transcription through its interaction with SREBP1 (Sterol Regulatory Element-binding Protein 1), influencing the production of IDH1 (Isocitrate Dehydrogenase (NADP(+)) 1) in glioma cells and their metabolic reprogramming [148].

SIRT7 has emerged as a critical regulator of mitochondrial function via its role in mitochondrial quality control. Under mitochondrial protein-folding stress (PFS^mt^), SIRT7 expression is induced, enabling SIRT7 to interact with nuclear respiratory factor-1 (NRF1) and become selectively recruited to the promoters of nuclear-encoded mitochondrial ribosomal proteins (mRPs) and mitochondrial translation factors (mTFs) [149,150,151,152]. Within this SIRT7/NRF1 axis, NRF1 stabilizes SIRT7 occupancy at these promoters; the loss of NRF1 markedly decreases SIRT7 binding, while SIRT7 knockdown derepresses mRP and mTF expression and increases mitochondrial biogenesis, respiration, and proliferation [151]. This transcriptional repression alleviates PFS^mt and limits mitochondrial translation, thereby reducing mitochondrial activity, tempering reactive oxygen species accumulation, and promoting cell survival under nutrient deprivation or proteotoxic stress [151]. In addition, SIRT7-mediated deacetylation of GABPβ1 enhances formation of the GABPα/GABPβ heterotetramer, facilitating coordinated expression of nuclear-encoded mitochondrial proteins and maintenance of mitochondrial homeostasis; its deficiency results in impaired oxidative phosphorylation and elevated oxidative damage, analogous to features of aging [149,150,152,153,154]. Collectively, these mechanisms underscore a mitochondria-directed role of SIRT7 in quality control, translational tuning of mitochondrial protein synthesis, and metabolic stress adaptation—all potentially relevant in CNS tumor biology.

## 9. Limitations

Our report refers to the U87 cell line, which, while widely used for studying glioblastoma, has several limitations that impact the reliability and applicability of the research findings. These limitations primarily stem from the genetic and phenotypic discrepancies between U87 cells and actual GBM tumors as well as the inability of U87 cells to fully recapitulate the complex tumor microenvironment and heterogeneity observed in human GBM [155]. These factors can lead to misleading conclusions and hinder the translation of research findings into clinical applications.

U87 cells exhibit a highly aberrant genomic profile, with numerous structural variations, single-nucleotide variations, and mutations that are not representative of typical GBM tumors [156]. For instance, the U87MG cell line has been shown to possess over 2.3 million single-nucleotide variations and 512 homozygously mutated genes, which may not accurately reflect the genetic landscape of patient tumors [156]. In vivo, the U87 cell line is known to form large, well-demarcated tumors, which contrasts with the highly invasive nature of GBM in patients [157]. This discrepancy can lead to an underestimation of the invasive potential of GBM and affect the evaluation of therapeutic strategies targeting tumor invasiveness [157]. Furthermore, U87 cells fail to capture the intra- and inter-tumoral heterogeneity that is characteristic of GBM [158]. This heterogeneity is crucial for understanding tumor behavior and response to therapies, as different cell populations within a tumor can exhibit varying sensitivities to treatment [158]. The U87 model does not adequately mimic the tumor microenvironment, including interactions with non-tumor brain cells, the extracellular matrix, and the immune system [159,160]. These interactions are essential for accurately assessing the efficacy of potential therapies and understanding tumor biology [159,160]. The limitations of U87 cells contribute to the high failure rate of GBM therapies in clinical trials, as preclinical models that do not accurately represent the disease can lead to false positives in drug efficacy studies [161]. The use of U87 cells may result in an incomplete understanding of GBM pathophysiology, which can impede the development of effective treatment strategies and the identification of novel therapeutic targets [162].

While U87 cells provide a convenient and cost-effective model for certain types of GBM research, their limitations necessitate the use of complementary models to obtain a more comprehensive understanding of the disease [149,152,153]. Emerging models, such as patient-derived neurospheres and organoids, offer promising alternatives that better capture the genetic diversity and microenvironmental interactions of GBM [152,153]. These models can provide more accurate insights into tumor biology and therapeutic responses, potentially improving the translation of research findings into clinical practice.

## 10. Conclusions

**Figure 3:**
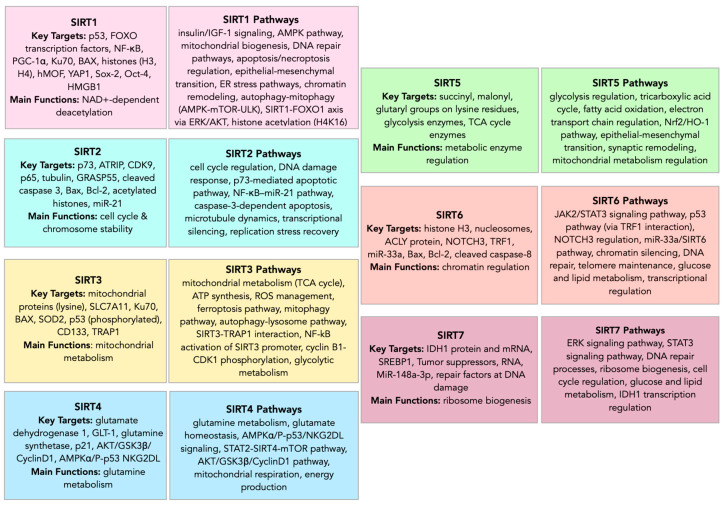
Sirtuin family—targets and pathways.

**Figure 4:**
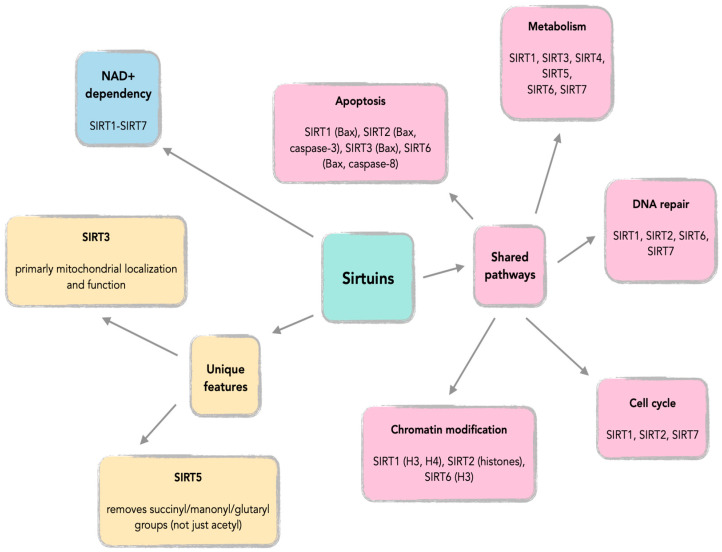
Common features across SIRT1-SIRT7.

**Figure 5:**
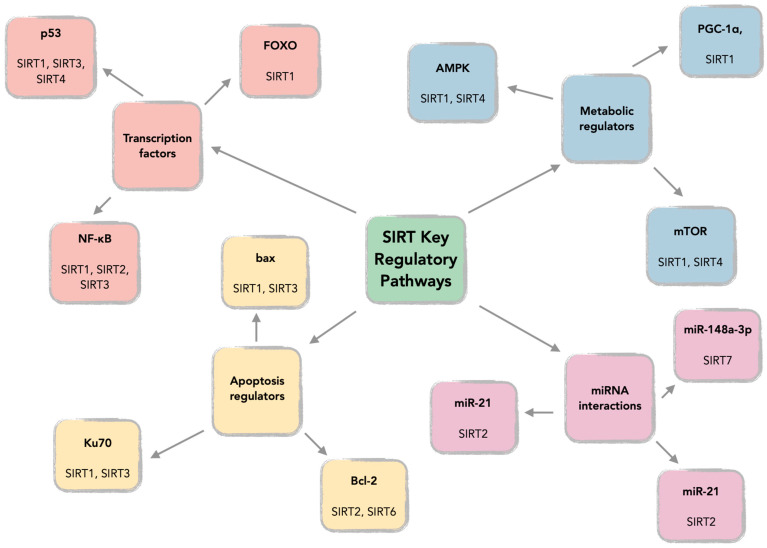
SIRT key regulatory pathways.

Sirtuins demonstrate complex, context-dependent roles in central nervous system tumors, exhibiting both oncogenic and tumor-suppressive functions (Figure 3, Figure 4, Figure 5, Table 1, Table 2, Table 3, Table 4). Their compartmental localization—nuclear, cytoplasmic, or mitochondrial—dictates their functional implications in glioma biology and other CNS malignancies.

Over the last decades, the study of sirtuins in cancer biology has revealed their critical regulatory roles in tumor development and progression, influencing various biological pathways. Sirtuins can act as either tumor suppressors or activators, depending on the specific cancer type, stage, and microenvironment. Consequently, both sirtuin inhibitors and activators are under evaluation as potential cancer therapies. While many questions remain about the precise roles of specific sirtuins in cancer, the ongoing investigation aims to develop novel SIRT-targeted molecules for clinical use, especially given the limited success of current treatment strategies.

SIRT1 is frequently overexpressed in glioma tissues, and its aberrant cytoplasmic localization correlates with enhanced proliferation, invasion, and therapy resistance in glioma cells. Its inhibition not only reduces tumor growth but also sensitizes cells to temozolomide by increasing reactive oxygen species levels. Moreover, SIRT1 contributes to glioma stem cell maintenance and microglia-mediated tumor support, suggesting its pivotal role in tumor progression and resistance mechanisms.

Conversely, SIRT2 exhibits dualistic behavior. While some studies indicate its tumor-suppressive properties—via inhibition of miR-21 through NF-κB modulation—others show that its overexpression correlates with poor outcomes, particularly in ATRX-deficient gliomas. SIRT2’s deacetylation of p73 inactivates this tumor suppressor, thereby promoting tumorigenicity.

SIRT3 primarily functions in mitochondrial metabolism regulation and is significantly upregulated in glioblastoma tissues. It protects glioma cells from ferroptosis, contributes to therapy resistance, and is involved in maintaining glioma stem cell survival. These findings underline its pro-tumorigenic role in glioblastoma, although some studies suggest a tumor-suppressive effect in specific contexts.

SIRT4, largely associated with metabolic regulation, generally exhibits tumor-suppressive functions in CNS tumors. It has been shown to reduce glioma proliferation and influence glutamate metabolism, and it is significantly downregulated in glioma tissues, supporting its role as a negative regulator of tumor progression.

SIRT5, another mitochondrial sirtuin, appears to act predominantly as a tumor suppressor. Its downregulation is consistently observed in glioma and is associated with poorer patient prognosis. SIRT5’s regulation of mitochondrial metabolism and its involvement in synaptic remodeling further highlight its significance in glioma biology.

Nuclear-localized SIRT6 is notably downregulated in glioma and acts as a tumor suppressor through various mechanisms, including inhibition of the JAK2/STAT3 and NOTCH3 signaling pathways. Its overexpression leads to reduced proliferation, migration, and increased apoptosis in glioma cells. Moreover, SIRT6 is negatively regulated by miR-33a, forming a potential axis for therapeutic targeting.

SIRT7 is markedly overexpressed in higher-grade gliomas and contributes to cell proliferation, invasion, and chemoresistance through regulation of the ERK/STAT3 and IDH1-associated metabolic pathways. Its knockdown not only reduces tumor growth but also enhances the efficacy of temozolomide, positioning it as a compelling target in glioma therapy.

Collectively, these findings establish sirtuins as crucial regulators of CNS tumorigenesis, each exhibiting distinct and often contrasting functions depending on cellular context and tumor subtype. Their varied roles in metabolism, DNA repair, apoptosis, and cell signaling underscore the potential of sirtuin-targeted therapies in glioma and other CNS tumors. Future investigations are warranted to further delineate their mechanistic pathways and therapeutic implications.

## Figures and Tables

**Table 1:** Overview of sirtuin functions in CNS tumors.

Sirtuin	Molecular Mechanisms	Therapeutic Implications	Clinical Evidence
SIRT1	- Localized in nucleus and cytoplasm; structured with N- and C-terminal domains flanking a conserved catalytic core [10,11]. - Deacetylates histone and non-histone proteins (p53, FOXO, NF-κB, Ku70, PGC-1α) [16,18,33]. - Regulates chromatin remodeling, DNA repair, metabolism, apoptosis, oxidative stress, and inflammation [13,14]. - Modulates insulin/IGF-1, AMPK, and NF-κB pathways [19,22]. - Activity is NAD+-dependent and influenced by caloric restriction, resveratrol, fisetin, quercetin, and post-translational modifications [38,39].	- Mislocalized to the cytoplasm in glioma cells, altering proliferation and oxidative stress response [42]. - Inhibition via nicotinamide or siRNA reduces viability, invasion, and EMT; increases ROS levels [43,44]. - Targeted agents (EX-527, SRT2183, Comp 5, UA) induce apoptosis, ER stress, mitophagy, and autophagy [47,48,49]. - Regulates NF-κB; modulated by SENP1 and miR-376a [50,53]. - Promotes glioma stemness by regulating Sox-2, Oct-4, and gliomasphere formation [54,55]. - Enhances medulloblastoma aggressiveness; resveratrol reduces SIRT1 expression [56,57]. - NAD+ depletion through SIRT1 activation sensitizes IDH-mutant gliomas to treatment [58].	- Overexpressed in glioma and medulloblastoma tissues; associated with worse prognosis [43,56]. - Inhibition increases temozolomide sensitivity and reduces tumor growth in xenografts [43]. - PET/CT/MRI with 2-[18F]BzAHA visualizes SIRT1 activity and response to inhibitors [46]. - Comp 5 and UA significantly reduce tumor size in vivo [48,49]. - SIRT1 is essential for glioma stem-like cell survival and self-renewal [54,55]. - High expression found in aggressive medulloblastoma subtypes [56]. - SIRT1 activation inhibits growth of IDH1-mutant gliomas [58].
SIRT2	- Primarily cytoplasmic; contains a central catalytic domain flanked by flexible N- and C-terminal regions [59]. - Removes acetyl and long-chain acyl groups from lysine residues on histone and non-histone proteins [62,63]. - Regulates microtubule dynamics, mitosis, cell cycle, chromatin structure, transcription, metabolism, and genome stability [64,67]. - Involved in DNA damage response by deacetylating ATRIP and CDK9 [69,70]. - Modulates H4K20 methylation and Golgi structure [67,68]. - Activity influenced by phosphorylation and stress-related downregulation [59,73].	- Deacetylates and inactivates tumor suppressor p73 in glioblastoma, promoting cell survival and proliferation [71]. - Inhibition of SIRT2 reactivates p73 function and reduces glioblastoma tumorigenicity [71]. - Targeted in ATRX-deficient gliomas to reverse oncogenic transcriptional programs [72]. - Knockdown leads to increased histone acetylation, reduced motility, and induced senescence in ATRX-deficient cells [72]. - Overexpression decreases proliferation and induces apoptosis via cleaved caspase-3 and BAX; associated with miR-21 suppression [50]. - Inhibition leads to apoptosis and necrosis in C6 glioma cells [75]. - Modulates response to resveratrol in glioblastoma stem cells; required for cell cycle arrest effects [76]. - Inhibition in ATRT reduces stemness, promotes differentiation, and increases survival [77].	- Nuclear SIRT2 expression is higher in glioblastoma than in astrocytoma or healthy tissue; correlates with tumor grade and poor survival [74]. - Inhibitors reduce growth of ATRX-deficient glioma xenografts in vivo [72]. - SIRT2 overexpression linked to suppression of miR-21 and reduced glioma proliferation [50]. - SIRT2 inhibition in C6 glioma cells induces caspase-3-dependent apoptosis and necrosis [75]. - Resveratrol effects on glioblastoma stem cells are diminished when SIRT2 is blocked [76]. - SIRT2 inhibition reduces tumor growth and stemness markers in ATRT models; increases neuronal differentiation markers in vivo [77].
SIRT3	- Localized in mitochondria; NAD+-dependent deacetylase [78,79]. - Deacetylates mitochondrial enzymes, regulating the TCA cycle, ATP production, and ROS detoxification [36,78,82]. - Involved in oxidative stress response, inflammation, DNA repair, apoptosis, and autophagy [83,85,86]. - Expression regulated by nutritional state, physical activity, and environmental stressors [78]. - Modulates metabolic flexibility in tissues such as muscle and brain [87].	- Supports mitochondrial metabolism and survival in glioblastoma cells [89]. - Inhibition disrupts protein synthesis machinery and enhances RSL3-induced ferroptosis [90]. - Promotes mitophagy and regulates SLC7A11, influencing GSH homeostasis and resistance to oxidative damage [90]. - Downregulation by fraxinellone increases ROS, apoptosis, and reduces GBM progression [91]. - Promotes radioresistance in GBM via NF-κB-mediated transcription and CDK1-dependent phosphorylation [93]. - Maintains stemness in glioblastoma stem cells by regulating mitochondrial respiration and suppressing ROS [97,98]. - Stabilizes TRAP1 and enhances electron transport chain function under nutrient stress [98].	- Upregulated in glioma patients; linked to tumor aggressiveness and survival [90,94]. - Inhibition sensitizes GBM cells to ferroptosis in vitro and in vivo [90]. - Fraxinellone reduces tumor size and SIRT3 signaling in vivo [91]. - SIRT3 overexpressed after radiation; required for mitochondrial function and resistance [93]. - SIRT3 highly expressed in glioblastoma stem cells; associated with CD133^+^ phenotype and metabolic adaptation [97,98]. - SIRT3 knockdown increases radiation sensitivity and disrupts GSC survival [93,98].
SIRT4	- Localized in mitochondria; exhibits ADP-ribosyltransferase and lipoamidase activity [99,100,101]. - Regulates glutamine metabolism by inhibiting glutamate dehydrogenase (GDH) through ADP-ribosylation [100,103,104]. - Controls fatty acid oxidation, ATP production, and redox balance [100,102,103,104,109]. - Functions as a tumor suppressor in various cancers by restraining mitochondrial metabolism and proliferation [100,103,108]. - Expression induced by DNA damage; modulates mitotic fidelity and cellular senescence [99].	- Acts as a tumor suppressor in glioma by inhibiting glutamine metabolism and reducing mitochondrial activity [94,111]. - Overexpression reduces glioma cell viability, proliferation, migration, and colony formation [94,111]. - Promotes neuronal apoptosis via the STAT2-SIRT4-mTOR [106]. - Enhances sensitivity of glioma cells to temozolomide when overexpressed [94,111]. - Inhibits AKT phosphorylation and reduces glycolytic activity [103].	- Downregulated in glioma tissues compared to normal brain [94]. - SIRT4 overexpression in glioma cell lines suppresses tumor characteristics in vitro [94,111]. - Shows significant downregulation in glioma patients compared to healthy controls, suggesting tumor-suppressive potential and value as a biomarker [94]. - Low SIRT4 expression is associated with shorter survival times in neuroblastoma patients; overexpression reduces proliferation, invasion, migration, and mitochondrial respiration [107].
SIRT5	- Localized in mitochondria; also detected in cytosol and nucleus [112,113]. - Exhibits weak deacetylase activity but strong desuccinylase, demalonylase, and deglutarylase activity [113,115]. - Regulates the urea cycle, fatty acid β-oxidation, ketogenesis, ROS detoxification, and TCA cycle via deacylation of mitochondrial enzymes [116,117]. - Modulates metabolism under fasting or stress conditions and contributes to redox homeostasis [116,117]. - Role in synaptic remodeling and neuroplasticity [8].	- Acts as a tumor promoter or suppressor depending on context [8]. - Suppresses tumor growth in gliomas by regulating mitochondrial metabolism [8].	- Lower SIRT5 expression in glioblastoma tissues compared to normal tissues [119]. - Lower SIRT5 correlates with shorter survival in GBM patients; negatively correlated with DNA methylation [119]. - Significantly downregulated in glioma patients vs healthy controls, suggesting tumor suppressor role [94,119]. - Low SIRT5 may promote cancer progression through increased ROS and metabolic disturbances [94].
SIRT6	- Crucial for chromatin silencing and transcriptional regulation [129,130]. - Regulates energy metabolism by suppressing glycolysis and protects retinal ganglion cells from oxidative stress [123]. - Regulates DNA repair, telomere maintenance, and glucose and lipid metabolism, maintaining cellular homeostasis [124]. - Modulates histone acetylation states, influencing chromatin structure and gene expression, especially in cancer [129,132]. - May localize to cytoplasmic stress granules in response to stress and aid recovery [133]. - Overexpression induces apoptosis via translocation of apoptosis-inducing factor from mitochondria to nucleus and reduces oxidative stress by lowering ROS and malondialdehyde, increasing antioxidant enzyme activity [135].	- Exogenous substances such as resveratrol, sirtinol, flavonoids, cyanidin, and quercetin can affect SIRT6 expression levels [128]. - Overexpression of SIRT6 in glioma cells reduces proliferation, migration, invasion, and increases apoptosis [45,135]. - miR-33a directly targets SIRT6, decreasing its mRNA and protein levels [136,137]. - SIRT6 counteracts effects of TRF1 knockdown in GBM cells, helping maintain cell viability and proliferation [134]. - SIRT6 induces apoptosis by increasing BAX and cleaved caspase-8 and decreasing Bcl-2 [136]. - Inhibits JAK2/STAT3 signaling pathway often overactive in gliomas [135,136]. - Negatively regulates NOTCH3 expression, reducing tumor-promoting effects [45].	- Downregulated in human glioblastoma multiforme tissues compared to adjacent non-tumor tissues, indicating a tumor suppressor role [135]. - Downregulated in glioma tissues and cell lines compared to normal brain tissues, associated with glioma development [45]. - Knocking down SIRT6 increases glioma cell proliferation, migration, and invasion [45]. - Inverse correlation between miR-33a and SIRT6 levels in glioma tissues; low SIRT6 correlates with tumor aggressiveness [136]. - TRF1 knockdown decreases SIRT6 expression via p53 pathway, potentially contributing to aging and autophagy [134].
SIRT7	- Located primarily in the nucleolus, involved in ribosomal biogenesis and cellular stress responses [139]. - Maintains genome stability by DNA repair and chromatin regulation; recruits repair factors to DNA damage sites [140]. - Regulates ribosome biogenesis and metabolic processes including glucose and lipid metabolism [141]. - Modulates target proteins in adipose and liver tissues, affecting metabolic diseases like type 2 diabetes and obesity [141]. - RNA binding enhances SIRT7’s catalytic efficiency and ability to remove long-chain fatty acyl groups [144].	- Regulates IDH1 expression and metabolic pathways in glioma cells via interaction with SREBP1, impacting α-KG levels, lipogenesis, and gluconeogenesis [148]. - Downregulation reduces glioma cell proliferation and invasion via inhibition of ERK and STAT3 pathways [146]. - Knockdown enhances cytotoxic effects of TMZ, suggesting SIRT7 mediates TMZ resistance [147]. - Targeting SIRT7 may improve chemotherapy efficacy in glioma [146,147]. - MiR-148a-3p regulates SIRT7 expression. Its decreased levels in glioma contribute to progression and therapy response [147].	- SIRT7 is highly expressed in human glioma tissues, especially in higher-grade tumors, correlating positively with glioma malignancy [146]. - Expression is significantly upregulated in glioma tissues and cells and correlates with pathological grade and poorer survival [147]. - In mouse xenotransplantation models, SIRT7 knockdown inhibits tumor growth and enhances TMZ antitumor effects [147].

**Table 2:** Functional categorization of sirtuins in CNS tumorigenesis.

Sirtuin	Functional Category	Primary Role in CNS Tumors	Tumorigenic Role	Mechanisms and Molecular Functions
SIRT1	Epigenetic Modulator, Metabolic Regulator	Maintains glioma stemness, represses tumor suppressors, enhances cell survival	Oncogenic	- Localized in nucleus/cytoplasm; deacetylates histones and non-histone targets (p53, FOXO, NF-κB) [16,33]. - Represses pro-apoptotic genes, enhances stress resistance, promotes EMT [43,44]. - Overexpressed in glioma tissues compared to normal brain tissue; higher expression correlates with poorer overall survival [43,44]. - Promotes epithelial-mesenchymal transition (EMT) by increasing mesenchymal markers (fibronectin, vimentin) and decreasing epithelial markers [44,51]. - Supports chemoresistance and tumor growth; essential for glioma cell viability and invasion capabilities [43,44]. - Converts microglia into tumor-supporting cells through nuclear localization and hMOF deacetylation, reducing H4K16 acetylation [52]. - Regulated by miR-376a which targets SIRT1, leading to decreased YAP1/VEGF signaling and suppressed angiogenesis [53]. - Regulates stemness markers (Oct-4, Sox-2) in glioblastoma stem cells [54,55]. - Inhibition increases sensitivity to TMZ; overexpressed in GBM and medulloblastoma [43,56].
SIRT2	Epigenetic Modulator, Cell Cycle Regulator	Modulates microtubule dynamics and apoptosis; dual role in glioma depending on context	Context-dependent (Oncogenic/Tumor Suppressor)	- Cytoplasmic localization; deacetylates α-tubulin, p73, histone marks, and p65 at K310; central catalytic domain with flexible N and C termini subject to alternative splicing and phosphorylation [50,59,60,61,63,71]. - Deacetylates and inactivates p73 tumor suppressor in glioblastoma, promoting proliferation and tumorigenicity. Overexpressed in ATRX-deficient gliomas correlating with poor outcomes [71,72]. - Underexpressed in some glioma tissues. Overexpression decreases proliferation, induces apoptosis via cleaved caspase-3 and BAX upregulation, while downregulating Bcl-2 [50]. - Higher nuclear SIRT2 labeling index correlates with glioma malignancy and worse survival outcomes—patients with low SIRT2 expression have significantly longer survival [74]. - Regulates NF-κB-miR-21 pathway by deacetylating p65, blocking its binding to miR-21 promoter and suppressing miR-21 transcription [50]. - Essential for glioma cell survival. Reduction leads to both necrosis and caspase-3-dependent apoptosis in C6 glioma cells [75]. - Specifically expressed in glioblastoma stem cells (not normal neural stem cells). Mediates resveratrol effects on GSCs proliferation and cell cycle arrest [76]. - Critical target in SMARCB1-deleted atypical teratoid/rhabdoid tumors (ATRT)—inhibition decreases growth, clonogenic potential, stem cell frequency, and tumor-sphere formation while promoting neuronal differentiation [77]. - Inhibition in ATRX-deficient gliomas reverses transcriptional changes, increases acetylated histones, reduces cell motility, and promotes cellular senescence [72].
SIRT3	Metabolic Regulator	Supports oxidative metabolism and therapy resistance in GBM	Oncogenic	- Mitochondrial NAD+-dependent deacetylase; regulates TCA cycle, ATP production, ROS detoxification, and autophagy [78,79,80,81,82,85]. - Modulated by nutritional and environmental factors, regulated by small molecule activators [81,84,87,88]. - Overexpressed in glioblastoma (GBM), supports mitochondrial metabolism via lysine deacetylation [89,90]. - Promotes resistance to ferroptosis by modulating mitophagy and regulating SLC7A11 expression. Knockdown enhances RSL3-induced ferroptosis [90]. - Inhibition by fraxinellone increases ROS and apoptosis. Overexpression counteracts fraxinellone effects [91]. - Radiation induces SIRT3 expression via NF-κB. Phosphorylation (Thr150/Ser159) enhances mitochondrial function and radioresistance [93]. - Significantly upregulated in glioma, potential diagnostic/prognostic biomarker. Promotes tumor survival via Ku70-BAX interaction [94,95]. - Downregulated by linalool, disrupting SOD2 interaction and increasing oxidative stress. Overexpression reverses effects [96]. - Enriched in CD133^+^ glioblastoma stem cells. Regulates stemness via mitochondrial respiration, ROS control, and epigenetic control of CD133 [97]. - Stabilizes TRAP1 to support ETC and metabolic fitness in GSCs. Inhibition of SIRT3-TRAP1 interaction disrupts stemness and increases ROS [98].
SIRT4	Metabolic Regulator	Restrains glutamine metabolism, promotes autophagy and apoptosis	Tumor Suppressor	- Localized in mitochondria and centrosomes; regulates FAO, glutamine metabolism, insulin secretion [99,100,101,102,103,104]. - Overexpression reduces glutamate accumulation via GLT-1 and GDH upregulation, and GS downregulation [111]. - Protects glioma cells from kainic acid-induced toxicity [111]. - Downregulated in glioma and neuroblastoma; associated with worse prognosis [94,107]. - Modulates cell cycle and microtubule dynamics during G2/M phases [99]. - Suppresses tumor cell proliferation and energy metabolism [107].
SIRT5	Metabolic Regulator	Enhances metabolic reprogramming, oxidative stress resistance	Oncogenic	- Primarily localized in mitochondria; removes succinyl, malonyl, and glutaryl groups from lysine residues [112,113,115]. - Regulates glucose metabolism, TCA cycle, β-oxidation, and electron transport chain activity [116]. - Protects against oxidative stress and contributes to synaptic remodeling and stress responses [8,116]. - Downregulated in gliomas. Lower levels associated with reduced patient survival [94,119]. - Functional analyses link SIRT5 to epithelial–mesenchymal transition and intercellular signaling pathways [119]. - Knockdown enhances glioma cell growth, supporting its role as a tumor suppressor gene [8]. - Shows an inverse expression pattern to oncogenic SIRT3 [94]
SIRT6	Epigenetic Modulator, DNA Repair Coordinator	Inhibits glycolysis, maintains genome integrity, promotes apoptosis	Tumor Suppressor	- Nuclear; associates with chromatin, interacts with nucleosomes, and deacetylates histone H3 at K9 and K56 [120,121,122,129]. - Zinc-binding domain targets the acidic patch of histones via an arginine anchor; Rossmann fold facilitates DNA and NAD+ binding [120,122]. - Regulates gene silencing, glucose/lipid metabolism, DNA repair, telomere maintenance, and inflammation [121,123,124,125,126,127,128]. - Overexpression inhibits proliferation, migration, invasion, and induces apoptosis via mitochondrial AIF translocation and BAX/caspase-8 activation [45,135,136]. - Suppresses the JAK2/STAT3 signaling pathway and reduces oxidative stress markers (ROS, MDA) while increasing antioxidant enzyme activity [135,136]. - Negatively regulated by miR-33a; inverse correlation between miR-33a and SIRT6 levels in glioma [136]. - Decreased in GBM tissues and glioma cell lines; overexpression reduces NOTCH3 and counteracts TRF1 inhibition effects [45,134,135].
SIRT7	Epigenetic Modulator, DNA Repair Coordinator	Promotes proliferation and represses apoptosis-related pathways in glioma	Oncogenic	- Primarily nucleolar; contains conserved catalytic domain (D118, Y119, R120); interacts with RNA to enhance activity [138,144]. - Participates in DNA repair and chromatin regulation; maintains genome stability; regulates ribosome biogenesis and metabolism (glucose/lipid) [139,140,141]. - Acts oncogenically by destabilizing tumor suppressors and promoting proliferation via ERK and STAT3 signaling [142,143,146]. - Highly expressed in glioma tissues, especially in high-grade tumors (e.g., grade IV), correlates with malignancy and poor prognosis [146,147]. - siRNA knockdown reduces proliferation and invasion; enhances Temozolomide sensitivity in vitro and in vivo [146,147]. - Negatively regulated by miR-148a-3p, which is downregulated in gliomas [147]. - Regulates IDH1 expression and α-KG metabolism through interaction with SREBP1, contributing to metabolic reprogramming in glioma cells [148]."

**Table 3:** Sirtuin targets, pathways and functional outcomes in CNS tumors.

Sirtuin	Targets	Pathways	Functional Outcomes
SIRT1	- p53 [16,24,34] - FOXO transcription factors [16,33] - NF-κB [14,33,50] - PGC-1α [18,21,35,36] - Ku70 [22] - BAX [22] - HMGB1 [51] - hMOF [52] - YAP1/VEGF [53] - Sox-2, Oct-4 [54] - Histones (H3, H4) [15,16,17]	- NAD+-dependent deacetylation [19,22,37] - Insulin/IGF-1 signaling [19,22,33] - AMPK pathway [14,22,33] - Mitochondrial biogenesis [21,35,36] - DNA repair pathways [22] - Apoptosis/necroptosis regulation [23,24] - Epithelial-mesenchymal transition [44,51] - ER stress pathways [47] - Autophagy-mitophagy (AMPK-mTOR-ULK, PINK1-Parkin) [48] - SIRT1-FOXO1 axis via ERK/AKT [49] - Histone acetylation (H4K16) [52] - miR-376a targeting [53] - Chromatin remodeling [15,16,17]	- Aberrant cytoplasmic localization in glioma cells [42] - Highly expressed in glioma tissues vs. normal brain [43,44] - Promotes cell proliferation, invasion, and EMT [42,44,51] - Enhances chemoresistance to temozolomide [43] - Higher expression associated with poorer survival [43] - Maintains cancer stemness in glioma stem cells [54,55] - Converts microglia to tumor-supporting phenotype [52] - Dual role—some activators induce cell death via ER stress [47] - Regulatory role in glucose-mediated glioma malignancy [51] - Potential therapeutic target for IDH-mutant tumors [58] - Detectable by PET imaging for patient selection [46] - Context-dependent effects on oxidative stress response [42,43] - Higher expression in aggressive medulloblastoma subtypes [56] - Crucial for cancer stem cell survival and tumorigenicity [55]
SIRT2	- p73 (C-terminal lysine residues) [71] - ATRIP [69,70] - CDK9 [69,70] - p65 (K310 deacetylation) [50] - miR-21 [50] - Tubulin [60,61] - GRASP55 [67] - H4K20 methylation [69] - Cleaved caspase 3, BAX, Bcl-2 [50] - Acetylated histones [72]	- Cell cycle regulation [59,65] - DNA damage response [69,70] - p73-mediated apoptotic pathway [71] - NF-κB–miR-21 pathway [50] - Caspase-3-dependent apoptosis [75] - Microtubule dynamics [60,61] - Chromosomal stability and cell cycle regulation [69] - Transcriptional silencing [59,65] - Replication stress recovery [69,70]	- Promotes proliferation and tumorigenicity of glioblastoma cells [71] - Nuclear SIRT2 expression correlates with glioma malignancy and poor survival [74] - Overexpressed in ATRX-deficient gliomas, correlates with poor outcomes [72] - Inhibition reduces cell motility and promotes cellular senescence [72] - Acts as tumor suppressor when overexpressed—decreases proliferation and induces apoptosis [50] - Underexpressed in human glioma tissues and cell lines [50] - Essential for glioma cell survival—reduction leads to necrosis and apoptosis [75] - Specifically expressed in glioblastoma stem cells but not normal neural stem cells [76] - Mediates resveratrol effects on GSC proliferation and cell cycle arrest [76] - Inhibition decreases ATRT cell growth, clonogenic potential, and tumor formation [77] - Inhibition promotes differentiation and prolongs survival in animal models [77]
SIRT3	- Mitochondrial proteins (lysine acetylation) [89] - SLC7A11 [90] - Ku70 [95] - BAX [95] - SOD2 [96,98] - CD133 [97] - TRAP1 [98]	- Mitochondrial metabolism (TCA cycle, ATP synthesis, ROS management) [36,78,82,84] - Ferroptosis pathway [90] - Mitophagy pathway [90] - Autophagy-lysosome pathway [97] - SIRT3-TRAP1 interaction [98] - NF-kB activation of SIRT3 promoter [93] - Cyclin B1-CDK1 phosphorylation (Thr150, Ser159) [93]	- Significantly higher expression in GBM tissues compared to normal brain [90,94] - Protects GBM cells from ferroptosis [90] - Promotes cell proliferation, migration, and survival [91,95] - Enhances radioresistance in glioblastoma cells [93] - Maintains stemness in CD133+ glioblastoma stem cells [97] - Enriched in glioblastoma stem cells and promotes mitochondrial respiration [97,98] - Associated with advanced tumor grades and shorter survival [95] - Serves as potential biomarker for glioma diagnosis and prognosis [94,95] - Reduces oxidative stress and maintains mitochondrial function [96,98] - Inhibition increases sensitivity to ferroptosis and reduces cell viability [90,96]
SIRT4	- Glutamate dehydrogenase 1, GLT-1, Glutamine synthetase [100,103,104,111] - p21, AKT/GSK3β/CyclinD1 pathway components [103,104] - AMPKα/P-p53/NKG2DL pathway components [105] - STAT2-mTOR pathway components [106]	- Glutamine metabolism and glutamate homeostasis [103,104,111] - AMPKα/P-p53/NKG2DL signaling [105] - STAT2-SIRT4-mTOR pathway [106] - AKT/GSK3β/CyclinD1 pathway [103] - Mitochondrial respiration and energy production [107]	- Inhibits glutamine-dependent ATP production via glutamate dehydrogenase 1 ADP-ribosylation [103,104] - Provides protection against excitotoxicity by modulating glutamate metabolism and reducing glutamate accumulation [111] - Functions as tumor suppressor: reduces cell proliferation, invasion, and migration in neuroblastoma; downregulated in glioma patients [94,107] - Low expression correlates with advanced tumor stages, lymph node metastasis, and shorter survival times in neuroblastoma [107] - Reduces mitochondrial respiration and energy production in tumor cells [107]
SIRT5	- Succinyl, malonyl, and glutaryl groups on lysine residues [115] - Enzymes involved in glycolysis, tricarboxylic acid cycle, fatty acid oxidation, and electron transport chain [116] - Nrf2/HO-1 pathway components [8]	- Glycolysis, tricarboxylic acid cycle, fatty acid oxidation, and electron transport chain regulation [116] - Nrf2/HO-1 pathway [8] - Epithelial-mesenchymal transition and cell communication pathways [119] - Synaptic remodeling and neuroplasticity pathways [8] - Mitochondrial metabolism regulation [8]	- Functions as tumor suppressor: significantly downregulated in glioma and GBM tissues compared to normal tissues [8,94,119] - Higher SIRT5 expression correlates with more favorable prognosis in glioma patients [8] - Knockdown enhances glioblastoma cell growth, confirming tumor suppressor role [8] - Lower expression associated with shorter survival times in GBM patients [119] - Downregulation may lead to increased ROS levels and metabolic disturbances promoting cancer progression [94] - Negative correlation between SIRT5 expression and DNA methylation status [119]
SIRT6	- Histone H3 [129,130] - Nucleosomes [120,121,122] - ACLY protein [129] - NOTCH3 [45] - TRF1 [134] - miR-33a [136] - BAX, Bcl-2, cleaved caspase-8 [136] - Apoptosis-inducing factor [135]	- JAK2/STAT3 signaling pathway [135,136] - P53 pathway (via TRF1 interaction) [134] - NOTCH3 regulation [45] - miR-33a/SIRT6 pathway [136,137] - Chromatin silencing and transcriptional regulation [129,130] - DNA repair, telomere maintenance [124] - Glucose and lipid metabolism [124]	- Acts as tumor suppressor—inhibits glioma cell proliferation, migration, and invasion [45,135] - Induces apoptosis and increases cell injury [135,136] - Reduces oxidative stress and increases antioxidant enzyme activity [135] - Enhances sensitivity to oxidative stress-induced apoptosis [136] - Significantly downregulated in glioma tissues compared to normal brain [45,135] - Counteracts aggressive behavior induced by NOTCH3 [45] - Protects retinal ganglion cells from oxidative stress [123] - Regulates energy metabolism by suppressing glycolysis [123]
SIRT7	- IDH1 protein and mRNA [148] - SREBP1 [148] - Tumor suppressors [142] - RNA (enhances catalytic efficiency) [144] - MiR-148a-3p (regulatory microRNA) [147] - Repair factors at DNA damage sites [140]	- ERK signaling pathway [146] - STAT3 signaling pathway [146] - DNA repair processes [140] - Ribosome biogenesis [141,143] - Cell cycle regulation [143] - Glucose and lipid metabolism [141] - IDH1 transcription regulation [148] - Metabolic reprogramming [148]	- Highly expressed in human glioma tissues, especially higher-grade tumors [146] - Positive correlation with glioma malignancy and pathological grade [146,147] - Promotes glioma cell growth, proliferation, and invasion [146,147] - Mediates resistance to temozolomide treatment [147] - Knockdown reduces cell proliferation, invasion, and enhances TMZ cytotoxic effects [146,147] - Inhibits tumor growth in mouse xenotransplantation models [147] - Regulates cellular levels of α-ketoglutarate (α-KG) [148] - Impacts lipogenesis and gluconeogenesis in glioma cells [148] - Acts as oncogene by destabilizing tumor suppressors [142]

**Table 4:** Sirtuin modulators in glioma therapy.

Sirtuin	Modulator (Activator/Inhibitor)	Preclinical/Clinical Status	Potential Application in Glioma	Reference
SIRT1	Nicotinamide (Inhibitor)	Preclinical (in vitro/in vivo)	Inhibits glioma cell proliferation, increases sensitivity to oxidative stress and TMZ	[43,44]
SIRT1	EX-527 (Inhibitor)	Preclinical (imaging + in vivo pharmacodynamics)	Reduces SIRT1 activity, visualized via 2-[18F]BzAHA PET imaging	[46]
SIRT1	SRT2183 (Activator)	Preclinical (in vitro)	Induces ER stress, apoptosis and cell cycle arrest in glioma cells	[47]
SIRT1	Compound 5 (Activator)	Preclinical (xenograft model)	Induces autophagy, mitophagy and tumor suppression without toxicity	[48]
SIRT1	Ursolic acid (Activator)	Preclinical (mouse model)	Inhibits tumor growth via SIRT1-FOXO1 axis, reduces tumor size and weight	[49]
SIRT1	Resveratrol (Natural compound)	Preclinical (glioma and medulloblastoma models)	Modulates SIRT1 expression, affects stemness and growth	[54,56]
SIRT2	AK-7 (Inhibitor)	Preclinical (in vitro glioma cells)	Induces apoptosis and necrosis, decreases motility in C6 glioma cells	[75]
SIRT2	siRNA (Inhibitor)	Preclinical (glioma models)	Reduces motility and viability; induces senescence and apoptosis	[72]
SIRT3	Fraxinellone (Indirect Inhibitor)	Preclinical (mouse GBM model)	Downregulates SIRT3, increases ROS and apoptosis, reduces tumor growth	[91]
SIRT3	SIRT3 knockdown (Inhibitor)	Preclinical (IDH1-mutant and GSC models)	Sensitizes to ferroptosis and radiation, disrupts stemness	[90,93,98]
SIRT4	SIRT4 overexpression	Preclinical (glioma cell lines)	Enhances resistance to excitotoxicity, reduces glutamate levels, upregulates GLT-1 and GDH, downregulates GS	[110,111]
SIRT5	SIRT5 knockdown	Preclinical (glioma models)	Enhances glioblastoma cell growth, promotes tumor progression by disrupting mitochondrial metabolism	[8]
SIRT6	SIRT6 overexpression	Preclinical (glioma cell lines)	Reduces glioma cell growth and viability, induces apoptosis via AIF nuclear translocation, lowers ROS and lipid peroxidation, inhibits JAK2/STAT3 signaling	[135]
SIRT6	SIRT6 overexpression	Preclinical (glioma cell lines)	Suppresses proliferation, migration, and invasion, reduces NOTCH3 expression and reverses its tumor-promoting effects	[45]
SIRT6	miR-33a (Inhibitor of SIRT6)	Preclinical (glioma models)	miR-33a downregulates SIRT6, SIRT6 restoration increases ROS and LDH, promotes apoptosis	[136]
SIRT7	SIRT7 knockdown (siRNA)	Preclinical (glioma cell lines and xenografts)	Reduces proliferation and invasion, associated with reduced tumor growth	[146]

## Data Availability

No new data were created in this study.
